# Ancient genomes provide insights into family structure and the heredity of social status in the early Bronze Age of southeastern Europe

**DOI:** 10.1038/s41598-021-89090-x

**Published:** 2021-05-12

**Authors:** A. Žegarac, L. Winkelbach, J. Blöcher, Y. Diekmann, M. Krečković Gavrilović, M. Porčić, B. Stojković, L. Milašinović, M. Schreiber, D. Wegmann, K. R. Veeramah, S. Stefanović, J. Burger

**Affiliations:** 1 Laboratory of Bioarchaeology, Faculty of Philosophy, University of Belgrade, 11000 Belgrade, Serbia; 2 Palaeogenetics Group, Institute of Organismic and Molecular Evolution (iomE), Johannes Gutenberg-University Mainz, 55099 Mainz, Germany; 3 Department of Genetics and Evolution, Faculty of Biology, University of Belgrade, 11000 Belgrade, Serbia; 4 National Museum of Kikinda, Trg Srpskih Dobrovoljaca 21, 23300 Kikinda, Serbia; 5 Leibniz Institute of Plant Genetics and Crop Plant Research (IPK) Gatersleben, 06466 Seeland, Germany; 6 Department of Biology, University of Fribourg, 1700 Fribourg, Switzerland; 7 Swiss Institute of Bioinformatics, 1700 Fribourg, Switzerland; 8 Department of Ecology and Evolution, Stony Brook University, Stony Brook, NY 11790 USA; 9 Biosense Institute, University of Novi Sad, 21000 Novi Sad, Serbia

**Keywords:** Social anthropology, Evolutionary biology

## Abstract

Twenty-four palaeogenomes from Mokrin, a major Early Bronze Age necropolis in southeastern Europe, were sequenced to analyse kinship between individuals and to better understand prehistoric social organization. 15 investigated individuals were involved in genetic relationships of varying degrees. The Mokrin sample resembles a genetically unstructured population, suggesting that the community’s social hierarchies were not accompanied by strict marriage barriers. We find evidence for female exogamy but no indications for strict patrilocality. Individual status differences at Mokrin, as indicated by grave goods, support the inference that females could inherit status, but could not transmit status to all their sons. We further show that sons had the possibility to acquire status during their lifetimes, but not necessarily to inherit it. Taken together, these findings suggest that Southeastern Europe in the Early Bronze Age had a significantly different family and social structure than Late Neolithic and Early Bronze Age societies of Central Europe.

## Introduction

### Kinship studies in the reconstruction of prehistoric social structure

An understanding of the social organization of past societies is crucial to understanding recent human evolution, and several generations of archaeologists and anthropologists have worked to develop a suite of methods, both scientific and conceptual, for detecting social conditions in the archaeological record^1–4^. These methods have been used to investigate when social complexity, including social inequality, first appeared^5–8^, the nature and function of early forms of social stratification, and how these emergent structures were perpetuated over time and space^9–11^.

In the absence of written records, prehistoric social structure is reconstructed primarily via evidence from mortuary remains^12^. Archaeological kinship studies use mortuary evidence to understand the specific role of family structure in shaping social organization^13,14^, and are critical for determining how familial relationships have influenced the emergence of social complexity and the evolution of persistent inequality^5,10,15,16^.

The anthropological understanding of kinship embraces not only biological relatedness, but also a broad range of non-biological social relationships^12^. Recently, ancient DNA (aDNA) has been developed as a complementary line of evidence for reconstructing prehistoric kinship ties. Kinship is a highly variable concept and involves complex interactions between individuals in a society, of which ancient DNA can only elicit one facet: biological relatedness^17^. Only in combination with multiple lines of bioarchaeological and archaeological evidence can palaeogenetic data contribute to a comprehensive exploration of family concepts and social organization^18–23^.

### The development of vertical differentiation during the Early Bronze Age

There is little evidence for significant social stratification before the end of the Pleistocene^7^. It was the stable climatic conditions of the Holocene that enabled the adoption of sedentism and plant and animal domestication, stimulating massive increases in population growth^24^. This growth, in turn, affected the economic structure of prehistoric societies, eventually leading to even greater population pressure and changes in social organization^7,25^. From the Neolithic onwards, there appears to be a general trend of increasing social inequality^26^, which again intensifies significantly during the Chalcolithic and Early Bronze Age (EBA) and became visibly expressed in material culture and burial customs^22,27–30^. We note however that the traditional view of Bronze Age communities as hierarchical chiefdoms ruled by hereditary elites has been challenged by several authors, such that the extent and nature of social inequalities in Bronze Age communities are still a matter of debate (e.g.^31,32^).

The consensus is that economic innovations such as the long-distance exchange of ideas, knowledge, and "exotic" goods enabled significantly greater accumulation of material wealth, as well as the differential control over valuable resources. This increased social and economic complexity very likely supported the emergence of elite individuals^30,33,34^. EBA social complexity may have had biological relevance: in ranked societies, social status influences access to resources and positions of power, potentially of crucial importance for individuals' health and fertility^5,10^.

In southeastern Europe, there is evidence for the presence of social inequality in Late Neolithic communities^35,36^, and it seems that these inequalities increased or at least persisted until the EBA. A debate has arisen surrounding the mechanisms perpetuating status and wealth and it is hypothesized that in complex societies such as Bronze Age chiefdoms, kinship networks and affinal ties served as conduits through which the leadership distributed wealth, controlled labor, and reinforced its own status^30,34^. Anthropological studies suggest that lineage-based intergenerational transmission of wealth has been the main mechanism for persistence of wealth inequality^7,25^.

### The role of Mokrin in understanding EBA social organization

**Figure 1:**
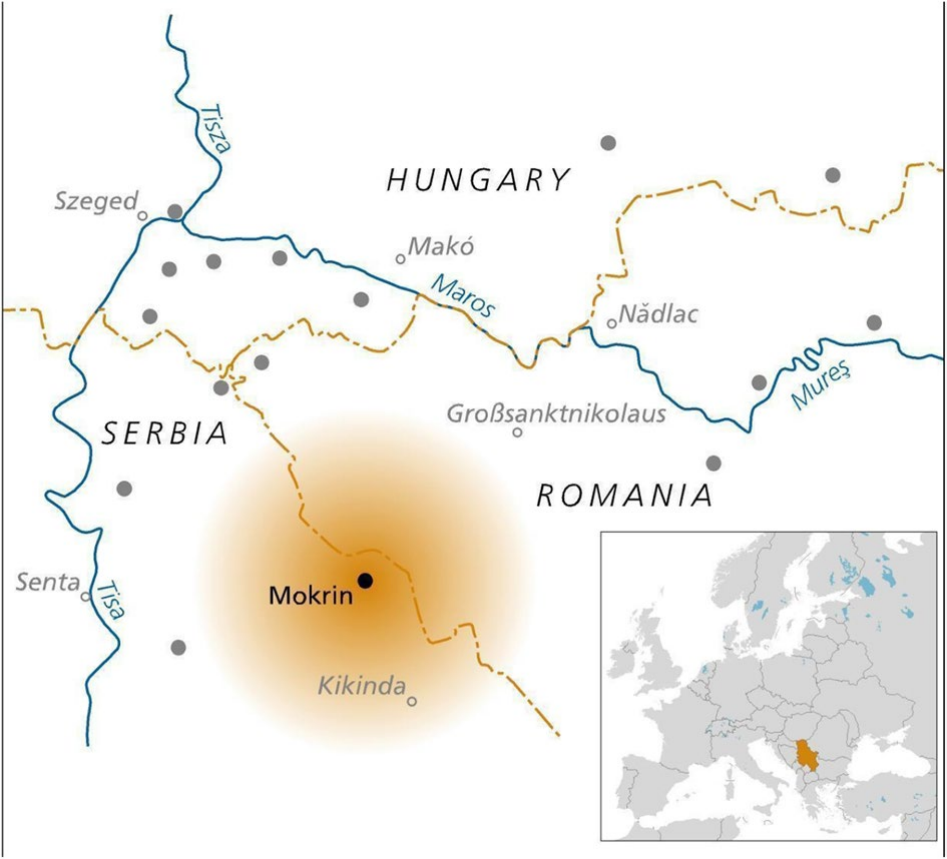
Location of the Mokrin necropolis in Southeast Europe. Dots represent other known Maros settlements and cemeteries.

Here we report the findings of a study combining palaeogenomic, bioarchaeological, and anthropological evidence to conduct a kinship analysis of the EBA necropolis of Mokrin, located near the town of Kikinda in the northern Banat, Serbia. The necropolis was used by a population belonging to the Maros culture (2700–1500 cal BC), which encompasses a set of communities extending through southeastern Hungary, western Romania, and northern Serbia^37^ (Fig. 1; Supplementary information 1). Radiocarbon dating indicates that the Mokrin necropolis was used for 300 years, from around 2100–1800 cal BC^38^, Supplementary Table S1). Large and well-preserved cemeteries are typical of Maros settlements, and Mokrin, with a total of more than 300 graves, is one of the largest. The normative funerary pattern in Mokrin consists of single inhumations. The bodies of the deceased were placed in a flexed posture, facing east, while the north or south body orientation varied by sex. While the females were placed on the right side with their heads oriented toward the south, the males were placed on the left side with their heads oriented toward the north^37,39^ (Supplementary information 2). In this study we rely on the not entirely unfounded assumption that biological sex was not fully disconnected from social gender in Mokrin and refer to the studies by Rega (2000)^40^, Porčić (2010)^41^ and Matić (2012)^42^ for further details.

The siting of Maros villages along rivers and the presence of distinctively non-local grave goods indicate that Maros communities engaged in frequent interactions with non-local groups, including the exchange of manufactured goods and resources^39^. The bioarchaeological and material-cultural correlates of social organisation at Mokrin have already been well characterised through a detailed paleodemographically informed analysis of grave goods in terms of their quantity, quality, and spatial distribution^39^ (Supplementary information 2). Further studies have investigated the ways in which physical activity patterns, discerned from skeletons, reflect wealth, status, and socio-political factors^43^. These lines of research have identified grave goods that functioned as markers of higher social status^39^; at the same time they detected a positive correlation between these markers and increased male physical activity^43,44^ (Supplementary information 1; Supplementary Figure S1). Undoubtedly, grave goods are not perfect reflections of the social realm, and can carry multiple meanings^45,46^. Furthermore, grave inventories are never complete, especially since items made of perishable material cannot be recovered. Nevertheless, previous research done on Mokrin necropolis^39,43^ indicate that recovered grave goods do reflect important social dimensions and that they can function as a rough proxy of social status, at least. The demonstration that grave goods are regularly, if not linearly, related to the social existence of an individual forms the explicit basic assumption of our following investigation into the heritability of social status in Mokrin.

In this study, we conducted palaeogenomic analyses on 24 skeletal samples from the Mokrin necropolis to provide unambiguous identification of biological relatedness between individuals. Identified biological relationships are then used together with archaeological markers of social status to infer features of burial customs, family structure and status transmission within a EBA Maros society, and—more broadly—to trace aspects of the development of Bronze Age societies.

We address four main questions:

1. What was the kinship system at the community served by the Mokrin burial site: were families organized in clans, lineages, or larger kindreds, and can we reconstruct residence and marital patterns?
2. Were wealth and status hereditary in the society represented by the Mokrin assemblage?
3. Does the genetic variability in the Mokrin sample correspond to that of a single population? Is there evidence of inbreeding?
4. How is the Mokrin assemblage representative of the genetic diversity during the Copper Age–Bronze Age transition?

## Results

### Sampling and anthropological analysis

**Figure 2:**
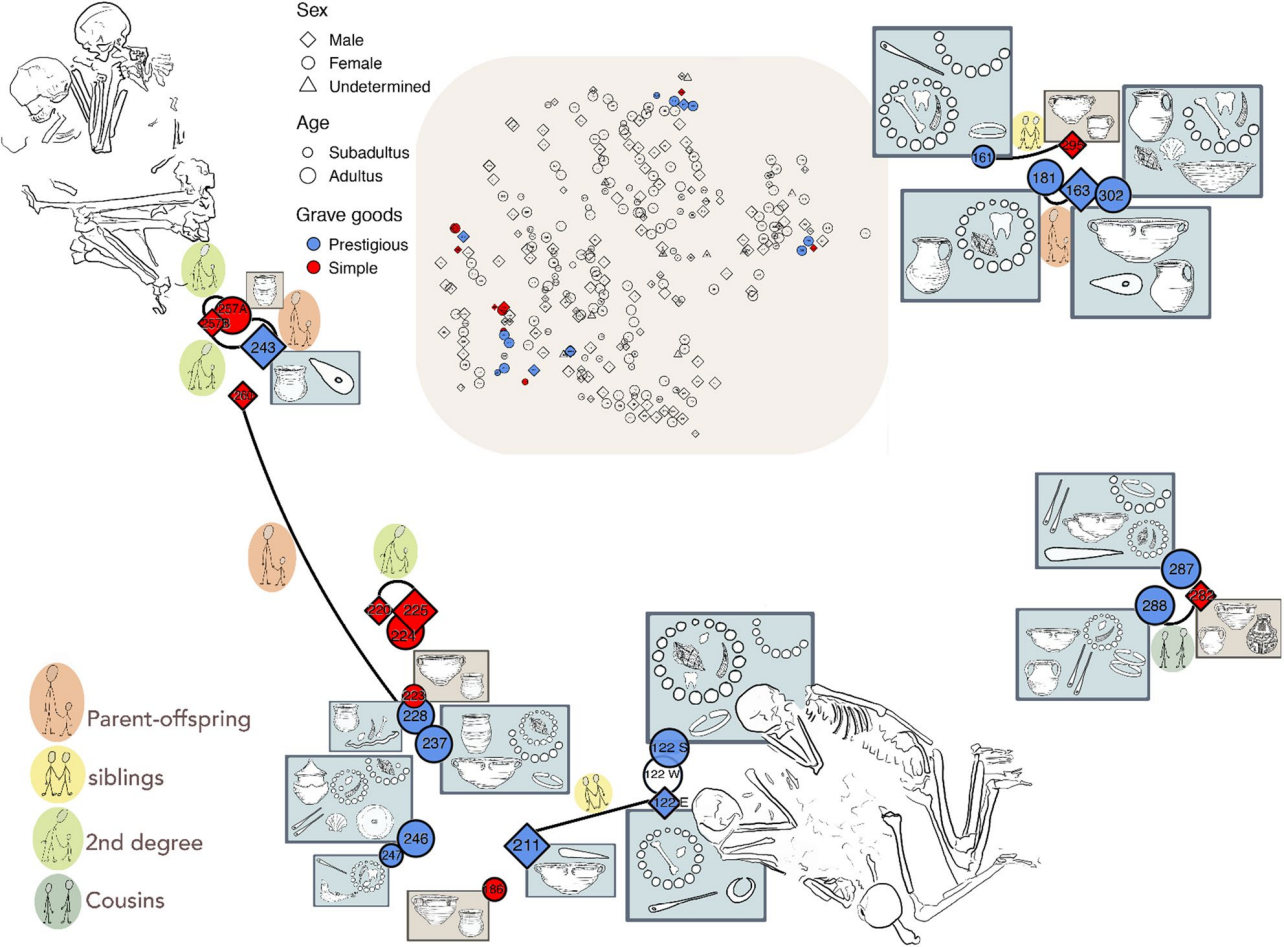
Visual representation of the 24 analysed individuals and their relationships at the Mokrin necropolis. Head ornaments are represented by semi-circles, necklaces by closed circles, and beaded sashes by wavy lines. The material used for making the jewellery is indicated by the symbol inside the jewellery. The colours reflect whether the burial equipment was classified as prestigious (blue) or simple (red).

We selected 24 individuals (14 adults and 10 children) buried in 22 graves for analysis according to a suite of criteria including petrous bone preservation, the presence of neighbouring graves, the presence of younger individuals buried in close proximity to adults (as potential family groupings to track the inheritance of status), and variety in material culture markers (Table 1, Fig. 2, Supplementary information 2). We primarily sampled 20 individuals from single graves and two individuals each from a double-burial (257) and a triple-burial (122; the third individual was not analysable due to poor preservation) (Supplementary Figure S2). Palaeopathological and physiological stress markers observed are typical for a prehistoric population of this area and period (See Supplementary information 1 for more details, Supplementary Table S2).

**Table 1:** Early Bronze Age Mokrin samples analyzed: burial information, genetic sex determination, age, grave goods, genomic sequencing coverage and uniparental haplotype information.

Burial	Genetic sex	Age	Grave goods	X-fold genomic depth	mt haplotype	Y haplotype
122E	XY	6–9	bone needle, kaolin and bone bead necklace, bronze earring	1.09	U5a2b1a	I2a1b
122S	XX	35–50	*Columbella* shells, *Dentalium*, animal teeth and kaolin necklace, bronze bracelet, bronze head ornament	0.78	H32	*
161	XX	9–11	necklace consisting of kaolin and *Dentalium* beads, and animal bones and teeth, bronze head ornament, bone needle, bronze ring	1.20	H80	*
163	XY	45–55	biconical vessel, stone hammer-ax, biconical beaker	1.21	U4a2	J2b
181	XX	> 18	biconical beaker, necklace made of *Dentalium* snail, deer tooth, kaolin and an oblong plaque of copper sheet	0.62	U4a2	*
186	XX	8–11	biconical beaker, biconical vessel	0.33	H1aj	*
211	XY	50–55	bigger bowl, bronze dagger	0.79	U5a2b1a	I2a1b
220	XY	15–25	-	0.64	T2b11	R1b1a2a2c1
223	XX	7–10	Beaker biconical vessel	0.39	U3a1	*
224	XX	25–40	–	0.77	T2b	*
225	XY	25–35	–	0.82	J1b1a1	R1b1a2a2c1
228	XX	35–50	ball-like beaker, beaded sash (bone pearls, a bead made of *Dentalium* shell, a kaolin clay spindle whorl)	0.95	J1c	*
237	XX	15–20	bronze head ornament, kaolin and *Dentalium* necklace, bronze bracelet, biconical bowl, biconical amphora	0.89	T2b	*
243	XY	20–35	biconical beaker, stone axe	1.12	H	BT
246	XX	45–50	Amphora, lid, head ornament consisted of bronze plaques, *Columbella* shell and pendants, necklace made of bronze bead, kaolin and *Dentalium* snail, Unionidae freshwater mussel, two bone needles	0.98	H80	*
247	XX	10–12	kaolin, *Dentalium* and animal teeth necklace, bone needle, sheep jaw	0.90	H1	*
257 A	XX	40–60	-	0.60	H	*
257 B	XY	inf. I	biconical beaker	0.61	K1a4	R1b1a2a2c1a1
260	XY	15–18	-	0.92	J1c	I2a2a1a2a2
282	XY	15–20	biconical beaker, bellied beaker with specific pattern, biconical bowl	1.41	H2b	BT
287	XX	20–35	bronze head ornament, copper chisel (possibly used for trepanation), two bone needles, copper bracelet, necklace made of *Columbella* shell and *Dentalium*, copper and kaolin beads, gold pendant, two biconical vessels	0.81	U5b2a2c	*
288	XX	60 +	two copper bracelets, necklace made of *Dentalium* and kaolin beads, two bone needles, biconical bowl, biconical beaker	0.81	HV0e	*
295	XY	15–20	cylindrical cup, biconical bowl	0.82	H80	I2a1a
302	XX	20–35	Beaker, conical bowl, copper head, ornament with kaolin, animal teeth and bones, *Dentalium* beads, *Columbella* shell and *Cardium sp.*	0.89	J1c	*

Based on the results of the analysis of grave goods carried out by O’Shea in 1996^39^ (described in Supplementary information 1), we divided our sample into two categories according to the richness of their grave goods: ‘prestigious’—characterized by the presence and number of high status-indicating grave goods; and ‘simple’ burials, i.e. those having few or simple grave goods or none at all.

### Assessment of DNA-preservation, contamination, post-mortem damage, and sex

We extracted DNA from petrous bones and performed whole genome shotgun sequencing, reaching an average depth of 0.85X ± 0.25 (0.33X−1.41X) on the autosomes (Table 1). The proportion of endogenous human DNA in the 24 investigated skeletons ranged from 8 to 70% (with only two samples below 20%), reflecting the very good molecular preservation of these samples (Supplementary information 3, Supplementary Dataset 1). Excellent preservation was also indicated by the low levels of contamination detected in the samples: the average mitochondrial contamination level was < 1% as estimated by contamMix (Supplementary Dataset 1)^47^. For non-USER treated libraries, deamination rates ranged from 0.13 to 0.26 at the first base of the 5′ end of the reads (Supplementary information 3, Supplementary Dataset 1), supporting the authenticity of the aDNA data.

Fourteen females and ten males were identified via molecular sexing^48^ (Table 1), confirming the anthropological sexing of the remains. Even though genetic sexing results for the individual from grave 211 were initially inconclusive following Skoglund et al. (2013)^48^, the amount of reads mapping to the X and Y chromosomes respectively indicated a XY karyotype, a result consistent with the morphological examination. This was further confirmed by applying the method described in Cassidy et al. (2020)^49^. We observed three discrepancies between molecular and archaeological sex assignment, all in individuals whose initial sex determination was based on the assumptions of the standard Mokrin funerary ritual alone (122S, 220 and 257B)^37^. Two of these cases (257B and 122S) are from a double and a triple grave respectively, which are from an archaeological perspective out of the norm, so the body orientation was probably affected as well.

### Uniparental markers and genomic diversity estimates

The Mokrin sample displays relatively high haplogroup diversity for both the non-recombining portion of the Y-chromosome (NRY, ĥ = 0.81) and mitochondrial DNA (mtDNA, ĥ = 0.95) (Table 1). We discerned at least 14 distinct mtDNA haplotypes, including several belonging to haplogroup U, often found in prehistoric Central European foragers^50^, as well as to H, T2, K1, and J1. The ten Y chromosomes could be assigned to at least five different haplotypes, of which three belonged to the R1b family common among modern European populations^51^.

No evidence of significant population genetic structure was found. We estimated the inbreeding coefficient* F* (defined as a deficit of heterozygote genotypes given population allele frequencies) to be zero, following the method described in^52^. We additionally tested several hypothesized partitions of our sample (Supplementary information 7), but did not observe significant population differentiation in any configuration (F_ST_ ≈ 0).

### Ancestry analyses

When projected onto a PCA of European populations, all Mokrin samples fall within modern European genetic variation, clustering in the midst of modern northern, eastern, and southern Europeans (Supplementary Figure S3).

We estimated individual admixture proportions under the assumption that the composition of a European Bronze Age population can be sufficiently well modeled with three components: Iron Gates hunter-gatherers, Aegean Neolithic farmers, and eastern European steppe-like populations. With possible exception of individual 186, we observed no significant variation in the eastern European steppe-like component between individuals (Supplementary Figure S4, Supplementary Dataset 2). Pooling individuals, admixture proportions are estimated to be around 12.5% (± 1.8% standard error (SE)) Iron Gates hunter gatherers, 53.7% (± 2.5% SE) Aegean Neolithic farmers, and 33.8% (± 2.3% SE) Eastern European steppe-like population (Supplementary Figure S5).

### Biological relatedness analysis

Genome-wide patterns of identity-by-descent among the 24 analysed individuals from the Mokrin necropolis revealed nine family relationships involving 15 Mokrin individuals (Table 2, Fig. 2). In addition to three parent–offspring and two sibling relationships, we reconstructed three second-degree (half-siblings, avuncular, grandparent-grandchild) and one third-degree (cousin) relationship. Inferred kinship relations were corroborated by an outgroup *f*_*3*_ analysis in which related individuals clustered together tightly (Supplementary Figure S6). Related individuals tended to be buried in close proximity (permutation test, p < 8.5 * 10^−4^), with two exceptions (Fig. 2). Nine out of 24 individuals did not have a close genetic relationship to any other individual (186, 122S, 223, 224, 237, 246, 247, 287, 302); they were all female (3 young girls and 6 adult women, 1.6 * 10^−3^). These women are evenly distributed over the entire sampling area (permutation test p < 1.6 * 10^−3^).

**Table 2:** Results of the kinship analysis obtained by lcMLkin software.

Biological relationship	Individual 1		Individual 2	
	Burial	Sex and age	Burial	Sex and age
**Parent–offspring relationship**				
	257A	♀, adultus	243	♂, adultus
	228	♀, maturus	260	♂, juvenis
	163	♂, adultus	181	♀, adultus
**Sibling relationship**				
	122E	♂, infans II	211	♂, maturus
	161	♀, infans II	295	♂, juvenis
**Second degree relationship (half-siblings, avuncular, grandparent-grandchild)**				
	257B	♂, infans I	257A	♀, adultus
	257B	♂, infans I	243	♂, adultus
	220	♂, juvenis	225	♂, adultus
**Cousin relationship**				
	282	♂, juvenis	288	♀, senior

### Phenotypic markers

We estimated frequencies of a set of markers related to pigmentation phenotypes in the Mokrin sample by calculating individual genotype likelihoods using a Bayesian approach implemented in ATLAS^53^. The frequency of the derived allele at rs16891982*G (*SLC45A2*) was 0.7098 (CI 0.5365–0.8476; N = 15) and at rs1426654*A (*SLC24A5*) 1 (CI 0.8899–1; N = 15); both are associated with skin depigmentation in Europeans^54^. Comparable frequencies can be found in modern day populations in Spain (*SLC45A2*: 0.8178, *SLC24A5*: 1). The frequency of the derived G allele at rs12913832 in the *HERC2* gene, which is strongly associated with iris depigmentation, was estimated to be 0.4498 (CI 0.2946–0.6127; N = 20), similar to modern day populations in Tuscany (0.4206; CI 0.4206 – 0.4415).

## Discussion

### The Mokrin population: ancestry, structure, and genetic diversity

The individual Mokrin genomes are well modelled as a mixture of Iron Gates hunter-gatherers, Aegean Neolithic farmers and influences from Eastern European steppe-like populations (mean *qpAdm* tail probability individually 0.42, pooled 0.07). The Aegean/Mediterranean ancestry component dominates in our sample (pooled 53.6% ± 2.5%), while the hunter-gatherer component is relatively low (pooled 12.5% ± 1.8%), and indeed it is statistically unsupported in five individuals.

The estimated inbreeding coefficient (*F*) is very low, suggesting that the necropolis, while inhabited by people with diverse ancestry, represents a randomly-mating population. We found a rather high number of mitochondrial lineages (14 haplotypes in 24 individuals). High mtDNA diversity in combination with archaeological and isotope evidence can indicate female exogamy^22,55,56^. Given the absence of genetic substructure and the fact that both Y-chromosomal and mitochondrial diversity is moderate-to-high at Mokrin, the most parsimonious explanation is that this cemetery served a single, large, and contiguous population.

### Biological relatedness and implications for the Mokrin kinship system

Genetic kinship analysis, which revealed close genetic relationships involving 15 of the 24 burials analysed, provided insight into the mortuary practices at the Mokrin site.

Our kinship analysis did not identify any fathers or daughters, and consequently, there were no mother-father-child trios. In contrast to a kinship study on Bronze societies in southern Germany^22^, we did not find genetic evidence for larger kindred, extended families, clans or lineages in our sample: this could be due to the limited number of samples analysed (24 out of 312 excavated burials), or insufficient power to reliably detect genetic relationships beyond the 3rd degree. We therefore cannot exclude the possibility that larger family units were buried scattered at the cemetery^57^. In any case, the funerary rite seems quite different from what is described in a BA sample from Germany^22^, where offspring were buried with both fathers and mothers.

In general, there is a trend in our sample that individuals who were buried close to each other are also genetically related. Even if this finding may be partly influenced by the sampling strategy, there is only a single case (grave 260 and 228) where close relatives (mother-son) were buried at a greater distance from each other.

All nine (ind. 186, 122S, 223, 224, 237, 246, 247, 287, 302) of the individuals from our sample with no genetic relatives among the Mokrin burials were female. Seven of them were found close to individuals with family ties suggesting that they may not have been entirely socially isolated in the local community. Their positions in the larger kinship networks of the Mokrin necropolis is difficult to infer with any degree of certainty. While it is possible that at least some of them were newcomers to the community, it is more likely that their relatives can be found in other, unsampled parts of the cemetery.

Marital relationship could be argued for two of them. Adult woman 224 was buried next to adult man 225, both without grave goods. A similar case is present with the young woman 302 who is buried close to the mature male individual 163. These two were buried close to the adult woman 181, who was probably the man's mother. All three had prestigious grave goods.

Taken together, we observe a tendency for individuals buried close to each other to be genetically related, but at the same time the presence of females with no biological relatives in close proximity. This suggests that the placement of burials in the necropolis was influenced by several mechanisms simultaneously – biological relatedness, social kinship ties, time of death, membership in social groups or social roles. This conclusion receives additional support from the case of the adult female 122S buried in a triple burial, who, contrary to the general trend, is not genetically related to the subadult 122E from the same grave.

The absence of detected biological daughters and the presence of women of different status with no kin in close proximity, considered together with the high mtDNA diversity observed in our sample, suggests that female exogamy may have been practiced in Mokrin’s source population. Female exogamy has recently been demonstrated for other regions of similar chronology in Southern Germany and Switzerland^22,56,58,59^ and apparently also played a role in more recent periods, such as the Early Middle Ages^60^.

While the above observations do not support an inference of strict matrilocality, we cannot definitively conclude that the Mokrin society was patrilineal and/or patrilocal. The observation that the necropolis was used by a single unstructured population in combination with a moderate to high Y-chromosomal haplotype diversity (h = 0.81) militates against strict patrilocality, as we then would expect to see a comparatively lower Y-chromosomal diversity, as in Mittnik et al. (2019)^22^ and Sjögren et al. (2020)^56^.

### Patterns of status transmission at Mokrin

Our sampling strategy targeted burials located close to each other within the Mokrin complex on the assumption that these would be more likely to contain genetically related individuals. Unravelling these biological ties through kinship analysis enabled us to explore whether wealth and social status (as indicated by grave goods) were inherited or earned in the Maros society represented by the Mokrin assemblage. If social prestige was transmitted intergenerationally, we would expect to see this reflected in status markers. Based on previous palaeogenomic and isotopic research from a similar period of prehistory^22,56^, and on previous anthropological and archaeological studies carried out on Mokrin^39,43,44^, we hypothesized that social status would be transmitted along patrilines, with women acquiring their status through marriage.

Although the differences in status within the Mokrin necropolis were not extreme, there were sufficient differences to distinguish between prestigiously and simply equipped graves (Fig. 2) and within the existing range there was even great variability in grave goods displayed by biological relatives. There were only two cases in which relatives expressed similar social status through their grave goods: the two men in burials 220 (15–25 years old) and 225 (25–35 years old) were second degree relatives and were both buried without grave goods—with a caveat that the young biological male buried in grave 220 was oriented S–N, which is a common orientation for females. At the opposite end of the status spectrum, a woman from the burial 181 was very likely the mother of the male in burial 163 and both were buried with grave goods indicative of higher social status^39^. Since the male individual was an adult at the time of death, it remains unclear whether he inherited or acquired his status.

Other observations at Mokrin do not support the inference that social status was transmitted intergenerationally to males. For example, the woman in burial 228 was the mother of the subadult male in burial 260 (15–18 years old). While the mother had comparatively rich grave goods, her son was buried without any grave goods at all. We infer that this subadult neither inherited his mother's social status nor acquired it in the course of his young life. Similarly, the woman 257A was identified as the mother of the 20–35 years old male buried in grave 243. Her grave goods suggest that she was of lower status, while her son’s grave contained an axe, an indicator of higher status. This status discrepancy suggests that the son acquired the status to command a richer burial during his life. We additionally note that the contrast in grave good richness observed in this quartet of burials is not consistent with the inheritance of status via the maternal line.

Evidence from another pair of burials demonstrates that sub-adults could have rich grave goods—if they were girls. A burial of a 9–11 years old girl (burial 161) contained various markers of higher status (a necklace, bronze head ornament, a bone needle, and a bronze ring). Her brother (burial 295), who was 15–20 years old when he died, had only small and simple ceramics beside him. The fact that these siblings display different status in the grave is more consistent with a system wherein females—but not males—could inherit social status. This case is particularly convincing evidence that only women could inherit status, as the girl was almost certainly too young to have had the opportunity to acquire status, and must have inherited her rich adornments, possibly as part of her dowry/bridewealth. However, an alternative explanation is that only very young children—but not teenagers/adolescents—inherited status in the grave. We further observed a cousin relationship between a higher-status adult female (288) and a lower-status juvenile male (282). The dissimilarity in status between these cousins is consistent with the idea that males did not inherit status, but does not rule out a system in which male children were assigned the status of their fathers once they reached a certain age or level of merit.

In total, we inferred biological kinship relationships for ten males in our sample. Given the observed status and age distribution, we can rule out that the higher–lower social status dichotomy in males is exclusively due to age differences in our sample. When we consider only first-degree relationships, for three out of six males it seems unlikely that status was inherited (burials 243, containing a 20–25 years old; 260, 15–18 years old; and 295, 15–20 years old), as their immediate female relatives (mother or sister) differed from them in their grave good status. For two other males (163, age 45–55; and 211, age 50–55), it is unclear whether status was inherited or acquired. The most problematic case for our interpretation is burial 122E, a 6–9 year-old boy buried with markers of higher social status—seemingly clear evidence of inherited status. However, 122E was part of the atypical triple burial and the assignment of the grave goods to the boy is not completely secure (see Supplementary information 2).

Taken together, these observations do not support the inference of inheritance of social status in men; male status appears to be acquired, except for the inconclusive case of the boy in grave 122. However, our sample does not include any of the fathers of the buried men and boys. We must therefore limit the scope of our claim to posit that sons in our sample have not inherited social status from their mothers.

### Reconstructing social organization at Mokrin in its temporal context

This analysis of 24 ancient genomes has illuminated important features of the social organization of the Early Bronze Age society served by the Mokrin necropolis, particularly concerning the inheritance of status. As already mentioned, the Mokrin skeletal sample appears to represent a genetically unstructured population. While this does not exclude the existence of social hierarchies, it does indicate that there were no strict barriers to marriage between social groups.

Multiple lines of evidence in our sample—high mtDNA variability, the presence of a certain number of unrelated women, and the absence of daughters—indicate that female exogamy was practiced between Mokrin and other settlements. Unrelated Mokrin females display a wide range of grave good richness, from poor to prestigious. Interestingly, the absence of adult daughters and presence of unrelated females has also been reported from Bronze Age farmsteads in Bavaria, where it was found that almost all unrelated non local females were buried in well furnished graves^22^.

At the Mokrin necropolis, relatives were buried close together in small kinship groups; interestingly, in our sample these small groups did not include biological fathers. The absence of larger kindreds and the relatively high Y-haplotype diversity in our sample are evidence against strict patrilocality in this population. At the same time, these observations suggest a different form of social organization from that of Bronze Age farmsteads in the Lech valley of southern Germany. Here, close relatives are also buried together, but there are clear signs of patrilocality^22,58^. There are also obvious differences to the Bell Beaker groups in southern Germany, an assemblage containing high mtDNA variability but only a single Y-chromosomal lineage (however, the low Y-variability here could be typical for the entire region at this time and have no social implications at all)^56^. And finally, the situation in Mokrin is also completely different from that in Late Neolithic families from Switzerland, where it has been found that male relatives are often buried together^59^.

Status inheritance at Mokrin also appears to have differed from other EBA cultures. Our kinship analysis identified three mothers, one sister, and nine unrelated females, but no daughters in our sample, complicating inference about inherited or acquired status in females. It seems that sons did not inherit social status from their biological mothers, but had the opportunity to acquire status throughout their lives. It is also possible that sons may have inherited their status from their fathers. An alternative explanation is that in Mokrin the law of the first-born was valid^56^ and in our sample only the post-born sons are present. In any case, the situation in Mokrin is certainly different from the Final Neolithic and EBA in Bavaria where clear signals of (male) status inheritance seem to be present^22,56^.

It is evident from the few existing palaeogenomic studies on this topic that there is significant regional and temporal variability in the social structures and heredity patterns of Late/Final Neolithic and EBA societies, although one common thread among the different societies investigated appears to be the practice of female exogamy. By illuminating the development of vertical differentiation and heredity systems, complete analyses of large cemeteries like Mokrin can help to trace the evolution of Early Bronze Age societies.

## Materials and methods

### Production of palaeogenomes

Sample preparation of petrous bones from 24 individuals was carried out in the aDNA laboratories of the Palaeogenetics group at the Johannes Gutenberg-University Mainz following the established protocol described in Supplementary information 3. Double-indexed libraries were prepared according to^61^ with modifications (see Supplementary information 3) and screened on an Illumina MiSeq platform at StarSEQ GmbH (Mainz, Germany). For deeper shotgun sequencing, aDNA extracts were treated with USER enzyme^62^ prior to library preparation. Whole-genome sequencing was performed on Illumina’s NovaSeq 6000 platform at the Next Generation Sequencing Platform (Institute of Genetics) at the University of Bern (Switzerland). For details see Supplementary information 3.

### Bioinformatic analyses

Raw data was analyzed with bioinformatics methods adjusted to account for the unique properties of aDNA, as described in detail in Supplementary information 4. Reads were aligned against the reference genome (GRCh37/hg19) using bwa aln^63^. During the conversion to the BAM format, reads were filtered for a minimal mapping quality of 30. PCR duplicates were marked using sambamba^64^ prior to realignment with GATK^65^ around known InDels.

Authenticity of DNA was assessed based on the mitochondrial chromosome using ContamMix^47^ and postmortem damage patterns (deamination at 5′ and 3′ ends) were quantified with MapDamage2 in aligned sequence reads for non-USER treated libraries^66^.

SNP calling was carried out following the approach described in^67^ using the ATLAS package^53^. Genotype calls were obtained with the maximum likelihood approach described in^67^. Majority-allele calls were produced for the SNPs overlapping the 1240 k capture array described in^68^, the Y-chromosome and the mitochondrial chromosome. In each case, sequencing errors and post-mortem damage were considered during variant detection. In addition, genotype likelihoods were calculated per individual and used for allele frequency estimates.

The molecular sex of each sample was determined following the approaches described in^48,49^. Y-chromosomal haplotypes were predicted with the yHaplo tool^69^, while mt-haplotypes were determined using Haplogrep 2.0^70^.

### Affinities and ancestry

For inferences about genetic affinities and ancestry, we computed a PCA with LASER v2.04^71^ against a reference space of modern European individuals^72^ and f-statistics with *qp3Pop*, *qpDstat* in *f*_*4*_ mode and *qpAdm* from the ADMIXTOOLS package^73^ (Supplementary information 5). *qpAdm* standard errors are computed by jackknifing excluding successive 5 cM blocks.

### Biological relatedness

Biological relatedness within the cemetery was estimated with lcMLkin^74^. The underlying assumption of these analyses is that close genetic relatives are more similar due to sharing alleles that are identical by descent because they were inherited from a recent common ancestor. Given the above genetic background, we performed genotype calling on the Mokrin samples at 6,191,202 SNPs that have a frequency of >  = 5% in the 1000 Genomes Eurasian samples^75^. We then performed biological relatedness estimation using three approaches on this set of SNPs (Supplementary information 6). All results were confirmed using pairwise distances and READ software^76^ using the original set of 6,000,000 SNPs, though READ was only able to identify relationships up to the second degree (Supplementary information 6). We assessed whether relatives tend to be buried close to each other using a permutation test. For this, we grouped the individuals into the four geographic groups north-east (287, 288, 282), north-west (257A, 257B, 243, 260), south-east (161, 295, 181, 163, 302) and south-west (220, 225, 224, 223, 228, 237, 246, 247, 186, 211, 122S, 122E) and quantified the number of pairwise relationships in which both individuals were buried in the same group. To assess significance, the observed number (eight) was compared against those obtained in 10^7^ permutations of the individuals among groups.

### Population genetic diversity estimates

The full sets of individuals we hypothesised may constitute clusters in a structured population are northern (161, 163, 181, 302, 287, 288, 243, 257A, 257B, 295, 260, 282) and southern (122E, 112S, 246, 247, 186, 211, 237, 220, 223, 224, 225, 228), individuals with (122S, 186, 223, 224, 237, 246, 247, 287, 302) and without (257A, 122E, 161, 163, 181, 211, 220, 225, 228, 243, 257B, 260, 282, 288, 295) family members buried in the necropolis, and an upper (181, 220, 287, 224, 225, 260, 228) and lower (all remaining) cluster in the PCA.

Pairwise distances were computed with PLINK^77^ (1-IBS distance) based on autosomal sites with one or two alleles called with ATLAS maximum likelihood caller^53^ as described above. The sets of individuals were filtered for relatedness, keeping only the genome with highest coverage in a family cluster of related individuals as given in Table 2, yielding the reduced set 122E, 122S, 163, 186, 220, 223, 224, 237, 243, 246, 247, 260, 282, 287, 295, 302. Comparisons between distributions of distances between groups were performed with a Kolmogorov–Smirnov Test as implemented in R.

We used the approach described in Burger et al. (2020)^52^ to determine the inbreeding coefficient *F*. Using ATLAS, a multi-sample vcf file was generated containing the genotype likelihoods of each individual. F was estimated based on genome-wide SNPs with a minimum quality of 40 that were covered at least twice in a minimum of ten samples. MCMC was run for 10^6^ iterations with ten burn-ins of 500 iterations each. In addition we re-ran the analysis while restricting F > 0.

Mitochondrial and Y-chromosomal haplotype diversity ĥ was estimated as:

$$\hat{h} = {n/\left(n - 1\right)} *(1 - \sum_{i=1}^{k} {\text{x}_{i}}^{2} )$$

where k is the number of haplotypes and x_i_ is the frequency of the i-th haplotype, estimated among n individuals.

### Allele frequencies of functional markers

Allele frequencies were estimated based on individual genotype likelihoods with ATLAS^53^ using a Bayesian approach. Frequencies were compared to European population samples from the 1000 Genomes project (CEU: Utah residents with Northern and Western European ancestry, GBR: British from England and Scotland, IBS: Iberian populations in Spain, TSI: Toscani in Italia) by F_ST_, using the Hudson’s estimator^78^.

## Data availability

Genomic data are available at the European Nucleotide Archive under the accession no. PRJEB38643 in BAM and fastq format.

## Supplementary Information

Supplementary Information 1.

Supplementary Information 2.

Supplementary Information 3.

Supplementary Information 4.
