# Supplementary material for: Ancient genomes provide insights into family structure and the heredity of social status in the early Bronze Age of southeastern Europe
Source: Sci Rep. 2021 May 12;11:10072. doi: 10.1038/s41598-021-89090-x (PMC8115322; doi:10.1038/s41598-021-89090-x)
Supplement: Supplementary file 1 — Supplementary Information 1. [file 41598_2021_89090_MOESM1_ESM.pdf]

Supplementary information for

Ancient genomes provide insights into family structure and the heredity of social status in the early Bronze Age of southeastern Europe

Aleksandra Žegarac, Laura Winkelbach, Jens Blöcher, Yoan Diekmann, Marija Krečković Gavrilović, Marko Porčić, Biljana Stojković, Lidija Milašinović, Mona Schreiber, Daniel Wegmann, Krishna R. Veeramah, Sofija Stefanović, Joachim Burger

Supplementary information 1. The Mokrin necropolis

Aleksandra Žegarac, Marija Krečković Gavrilović

The Early Bronze Age (EBA) necropolis of Mokrin (Serbia) is situated at the site of Lalina Humka, close to the town of Kikinda in the northern Banat, Serbia. It was a part of the Maros culture which represents a set of communities, spread out through the territories of southeastern Hungary, western Romania, and northern Serbia, between the Karas river at the North, Tisa river to the west and Zlatica and Galacka to the South[1]. Most archeological sites were found in vicinity to the Maros and Tisa rivers, and due to their similarity in the mortuary practice and material culture, it was suggested that these sites represent a cultural entity[2].

The Mokrin necropolis was systematically excavated from 1958 to 1965 under supervision of the National museum of Kikinda. In addition to 312 graves, which were excavated and published in great detail[1], some 50-100 graves remained unexcavated.

1.1. Dating

In total 24 radiometric dates are available from the Maros group, which flourished from around 2700 cal BC until 1500 years cal BC, with a possible peak around 2000 cal BC[3]. Six absolute dates from the Mokrin necropolis are in range from 2134 until 1737 cal BC, according to the uppermost and lowermost 1σ boundary of the oldest and most recent date, which settles the Mokrin cemetery into the first half of the Maros sequence and EBA[3] (Supplementary Table S1). This is in concordance with the relative chronology based on the typology of material culture[2]. The Mokrin necropolis was probably abandoned by the Middle Bronze Age period of the Maros sequence.

1.2. The settlements

Even though it was assumed that Maros communities lived in both open settlements and on hilltops that protected them from seasonal flooding[4], there is no evidence which suggests any differences in activities, functions or household architecture between these two settlement types[2]. Maros houses were relatively small rectangular dwellings, with clay floors, interior ovens and hearths, multiple rooms and large storage pits, and were probably home to single nuclear or small extended

families[2]. The locations of Maros villages in vicinity of rivers provided a good setting for regional trade networks and the movement of material (such as gold, copper, and tin ores).

Skeletal remains of domestic animals (horse, cattle, pig, sheep/goat, dog, etc) and wild mammals, such as red deer and beaver[5], suggest that inhabitants of Maros culture settlements were practicing agriculture, animal husbandry, hunting and fishing in everyday life. Maros inhabitants also domesticated barley and einkorn, plants of low yield but high resistance to wet and unpredictable areas of rivers[2]. They were active in cloth weaving, production of ceramics, and metallurgy[2].

1.3. The analysis of skeletal remains

The basic age and sex determinations of the human osteological sample were reported by Farkas and Liptak (1971)[6], while further physical anthropological analyses were conducted to investigate the biological status of individuals[4,7].

The results of the anthropological analysis[8] for 24 chosen individuals from the necropolis are presented in Supplementary Table S2. Even though the percentage of preserved bones in each grave was mostly fair, the taphonomic damage to the surface of the bone was high to severe, probably due to the high acidity of the soil. Due to the fragmentation of the long bones and previous analyses which involved cutting of the bones, it was not possible to determine approximate height[9] for half of the adult individuals (Supplementary Table S2). The teeth were fairly well preserved, but in some instances the enamel was compromised. Caries was present, but rare (five out of 24 individuals), while linear enamel hypoplasia appears to be slightly more common (exhibited in seven out of 24 individuals). Individuals with *cribra orbitalia, porotic hyperostosis* and *periostitis*, conditions fairly common in prehistoric skeletal assemblages, are listed in Supplementary Table S2. We also recorded an occurence of auditory exostosis (individual 228) and a case of fusion of the bodies of the L3 and L4 vertebrae (individual 246). The man buried in grave 225 had a possible healed depression fracture of the frontal bone.

1.4. Indicators of social status

Based on the demographic pattern, frequency and spatial distribution of specific grave goods, O'Shea (1996)[2] inferred different social status of individuals of the Mokrin cemetery and the Maros culture. Grave goods such as weapons (axes and daggers), head ornaments, bone needles and beaded sashes, are considered to be the markers of higher social status and connected with certain social or political positions. Their occurrence in burials varied depending on sex, age and location in the necropolis. The major social markers also shared several characteristics. They were inherited (except for head ornaments among females), or at least the right to wear them was inherited, as markers of status were also found in subadult burials. They were also limited to a smaller number of individuals, as there was probably a finite number of prestigious positions. In addition, major social markers were distributed within graves and the cemetery in a specific manner and mostly concentrated at one part of the necropolis[2]. The members of lower rank of the Mokrin necropolis were buried without any grave goods or only with simple ceramic and beads[4].

On the basis of the number of individuals buried with markers of higher social status, O'Shea (1996)[2] hypothesized a division of the Mokrin necropolis into two autonomous parts and the existence of two distinct communities using the

Mokrin cemetery, in which each community was connected and integrated through kinship ties, and in that manner managed to defend itself from conflict.

Although all Maros villages were autonomous, they were similar in mortuary rituals, material culture and local organization, inferring the same social or political organization in these communities[2].

Porčić and Stefanović (2009)[10] combined the archaeological and biological evidence to study connection between musculo-skeletal markers of physical activity and social status, and to answer the questions regarding the social structure of the Mokrin necropolis and to explore whether Mokrin was a ranked society. Although the basic hypothesis was that individuals of higher rank would be less physically active and vice versa, the results showed that intensity of activities was not related to the vertical status of an individual in a straight-forward way. There was an opposite correlation between social status and the development of shoulder and arm muscles when sex is observed separately, demonstrating that some rank was present. The males of higher status were more involved in upper body physical activities than males of lower rank, either due to a specialized activity related to their specific high position (e.g. weapon handling), or because they had to achieve their high status through the success in warfare or hunting, not through inheritance. When less physically active men were observed, there was a correlation with fewer grave goods. On the contrary, women of higher rank were less involved in a particular set of activities, while women with the most pronounced muscle attachments were buried without any grave goods. These findings can contribute to O'Shea's (1996)[2] hypothesis that women probably achieved their high status through their links with prestigious males and that the Mokrin society was not a highly ranked society, such as a chiefdom[2,10].

Supplementary information 2. Archaeological context of the samples

Aleksandra Žegarac, Marija Krečković Gavrilović

2.1. Inhumation

The predominant funerary pattern in Mokrin consists of single, primary inhumations. The bodies of the deceased were placed in oblong-shaped graves and positioned on their side in a flexed posture, facing east. In addition, the north or south body orientation and the side on which the body was placed varied by sex: the females were placed on the right side with their heads oriented toward the south and the males on the left side with their heads oriented toward the north[1]. Out of 73 skeletons determined as males by osteological analyses, 67 (92%) were oriented with their heads toward the north, while 102 out of 105 (97%) osteologically determined females were oriented with their heads toward the south[2]. There is only one individual whose position deviated from the north or south orientation, while seven individuals were facing the non-easterly direction. Apart from the possibility that these exceptions from the normative pattern in the Mokrin cemetery represent a distinct category of individuals, it is also plausible that sex was misidentified due to the availability of only cranial remains for identification.

The general alignment of graves at the Mokrin necropolis is along the north-south axis. Only one grave is oriented toward the west, while there is a series of graves with intermediate orientations. Apart from different grave alignment and body

orientation, there are also additional, alternative treatments documented at the Maros cemeteries, but not all of them are exhibited in Mokrin (Supplementary Table S3).

While there was no significant difference observed among adults in the depth of the graves, male graves were found to be significantly longer than graves of females. Child burials in general were less preserved, partly because of the sensitivity of the material, partly because of the smaller dimensions of the graves. Burials of children younger than three years were very rare at the Mokrin cemetery, which suggests that they had some alternative form of funerary treatment.

The alternative burial treatments in Mokrin also included multiple burials - several individuals sharing the same grave, in which most typically, a single adult (either male or female) and a single subadult were buried. In Mokrin, there are three double burials (graves 22, 257, 308) and one triple burial (grave 122)[1]. It is assumed that multiple burials did not necessarily have to represent a first-degree relationship (such as mother and child). The occurrence of joint burials raises questions about the biological relationship between the deceased and the cause of their simultaneous death. Since they were rare in the Maros culture, they may point to a significant departure from the normative custom or represent a very specific social category. It is possible that multiple burials reflected a practical treatment that was utilized in the event when a related adult and a subadult passed away at the same time or experienced specific circumstances of death linked to some dramatic event (accident, warfare, disease, etc)[2]. In addition, it was noticed that deviations from normative funerary practice exhibit a relatively even distribution across the site, as well as a certain co-occurrence of alternative treatments at the Mokrin cemetery. Thus, it is possible that these exceptions were intentional, representing graves of a distinct social category or mortuary status[2].

2.2. The material culture

The most common elements of Maros material culture are ceramics and copper and bronze artefacts. While coarse-ware was simple and probably manufactured in the context of individual households or communities, well made fine-ware ceramics (liquid containers, bowls, and biconical storage jars), with unique and uniform forms of containers, was probably manufactured in regional workshops, providing an evidence of local or regional exchange connections between the Maros villages[2]. Similarly, the two most likely sources for the metals were located at great distances from Maros villages[2]. Therefore, raw ores for production of standard Early Bronze Age weapons, such as daggers and axes, either had to be acquired via trade and smelted on the site, or already manufactured metal artifacts were brought to the villages. It is possible that some of the finished items derived from the Central European sources, while much of the raw material used in local manufacture derived from the Eastern sources[2]. Among the copper and bronze ornaments, standard Early Bronze Age forms were also found, such as neck rings, roll-headed and "Cypriot" arched pins, spiral arm rings, and finger and hair rings. Moreover, gold artifacts, such as hair accessories and finger rings made of coiled gold wire, were also associated with the Maros cemeteries[2].

Metal artifacts (gold, bronze and copper) were rarely encountered in Maros settlements and they appear to have been used more commonly for social display rather than for utilitarian purposes. Similarly, the objects obtained by trade, such as marine mollusks (*Dentallium* and *Collumbela sp.)* or Nagyrev ceramics are considered to have had a special value and represent direct evidence that people engaged in long distance travel[11]. O’Shea (1996)[2] provided a detailed analysis of the

variability of grave goods and demographic data, their quantity, quality and spatial distribution, and concluded that objects such as bone needles, head ornaments, weapons and beaded sashes were considered to be the markers of higher social status (Supplementary Figure S1). It is interesting that markers of higher status found with men were not produced locally, while women were buried with bone needles and beaded sashes of mostly local origin (kaolin, locally produced ceramics, animal bones and teeth)[2].

2.3. Investigated individuals

24 individuals, provided by the National Museum in Kikinda, were chosen according to age, sex, preservation of petrous bones, spatial distribution of graves, specific archaeological context and O'Shea's (1996)[2] social status criteria (Table 1). Based on the great variability of grave goods, 12 individuals (adults and children) from the Northern part of the necropolis were chosen, representing individuals of lower and higher status (graves number 161, 163, 181, 243, 257A, 257B, 260, 282, 287, 288, 295, 302). The same procedure and criteria were applied to the Southern part of the necropolis (graves number 122S, 122E, 247, 211, 237, 246, 224, 225, 228, 186, 220, 223). Among chosen individuals, there were two multiple burials: double burial 257 and triple burial 122, but without the third individual 122W, whose skeleton is too poorly preserved (Supplementary Figure S2).

Supplementary information 3. Production of palaeogenomes

Aleksandra Žegarac, Laura Winkelbach

All experimental procedures prior to PCR amplification (sample preparation, DNA extraction and library preparation) were carried out at the facility dedicated to ancient DNA analysis of the Paleogenetics Group, Institute of Organismic and Molecular Evolution (iomE) at the Johannes Gutenberg University, Mainz where appropriate protocols to prevent contamination with modern DNA were implemented[12]. All steps (pulverization, DNA extraction, library preparation and PCR reactions) included negative controls to monitor contamination and decontamination efficiency.

3.1. Sample preparation

For our analyses we collected the petrous bone, a part of the temporal bone, from 24 individuals from the Mokrin necropolis in cooperation with the National Museum of Kikinda, Serbia, as it is the densest bone in the body of mammals and contains the highest amount of endogenous DNA[13,14].

Prior to the analyses, meticulous documentation was carried out because of the destructive nature of aDNA analyses. The petrous bones were then sterilized under ultraviolet light (254 nm) from two sides for 45 min per side. The first step of sample preparation is removing superficial contamination and cleaning the bones including the canaliculi from the soil. Therefore, the outer surface of the bone was removed with a sandblasting machine (P-G 400, Harnisch & Rieth, Winterbach, Germany) using Spezial-Edelkorund (EW60/250 my and 30B/50 my; Harnisch+Rieth). A disk saw (Electer Emax IH-300, MAFRA) was utilized to obtain small, non-porous cubes of the densest, inner part of the petrous bones, which were irradiated again with

ultraviolet light (254 nm) for 45 min per side from two sides. The densest bone cubes were pulverized in a milling machine (MM200, Retsch) to obtain bone powder. Blank milling controls containing hydroxyapatite were processed in parallel and were treated as samples in all subsequent steps to control contamination. The bone powder is stored in a fridge until further processing.

3.2. DNA extraction

DNA was extracted following the protocol by Yang *et al.* (1998)[15] with the modification described in Gamba *et al.* (2014)[13] and MacHugh *et al.* (2000)[16] as well as additional modifications described below.

The extraction procedure included a pre-lysis step, to increase the amount of endogenous DNA and to eliminate contaminants. To each sample tube with 0.15 g of bone powder, 1 mL of EDTA (0.5 M, pH8, Ambion/Applied Biosystems, Life technologies, Darmstadt, Germany) was added and incubated at room temperature for 10 minutes. The solution was centrifuged at maximum speed to pellet the powder and the EDTA was removed carefully.

In the next step, 1 ml of extraction buffer containing EDTA (950 µl, 0.5 M, pH8, Ambion/Applied Biosystems, Life technologies, Darmstadt, Germany), Tris-HCl (20 µl, 1 M, pH8, Life Technologies, Carlsbad, United States), N-Laurylsarcosine (17 µl, 5%, Merck Millipore, Darmstadt, Germany) and Proteinase K (13 µl; 20 mg/ml, Roche, Mannheim, Germany) was added to the bone powder for lysis. Tubes were covered with parafilm, vortexed and incubated on rocking shakers at 37°C for 24 hours (900 rpm). After incubation, samples were spinned down for 10 minutes at 10,000 rpm, the supernatant was removed, transferred into new tubes (Extract 1) and stored in a fridge until further processing. If undissolved bone powder was left, a second lysis step was performed following the same procedure (Extract 2).

Following lysis, Extract 1 and Extract 2 were merged on an Amicon Filter (Amicon Ultra-4 30 kDA, 15 ml, Merck Millipore, Darmstadt, Germany) and centrifuged for 10 min at 2500 rpm. The DNA was then washed twice with 3 ml 1X Tris-EDTA, followed by centrifugation at 2500 rpm for 20 minutes and discarding of the flow-through in between. If needed, the final centrifugation step was extended until the sample was concentrated to 100 µl. Samples were subsequently purified with the QIAgen MinElute kit (Qiagen, Venlo, Netherlands) following the manufacturer's instructions with slight modifications during elution: 44 µl elution buffer (preheated to 65°C) were added to the columns and incubated for 5 minutes prior to centrifugation.

Blank controls were also processed and incorporated into all further steps of the analyses.

3.3. Library Preparation

To prepare DNA for Next Generation Sequencing (NGS) on Illumina platforms, double-indexed Illumina libraries were prepared according to the protocol by Kircher, Sawyer, and Meyer (2012)[17], optimized for ancient samples with slight modifications (Supplementary Dataset 1). For screening, the DNA extracts were not treated with USER enzyme as full damage patterns of DNA fragments were used to authenticate ancient DNA samples. Blank controls, as well as positive controls (nonsense hybrids) of known concentration, were processed in every library step and each PCR reaction to verify the success of the library preparation and to monitor contamination.

Blunt End repair was performed using the NEBNext End Repair Module (New England Biolabs, Ipswich, Massachusetts, United States): 20 µl of DNA extract are mixed with NEBNext End Repair Reaction Buffer (10X, 7 µl), NEBNext End Repair Enzyme Mix (3.5 µl) and nuclease-free water (39.5 µl; for a final reaction volume of 70 µl) and incubated for 15 minutes at 25°C followed by 5 minutes at 12°C. In the adapter ligation step hybridized adapters P5 and P7 (IDT, Leuven, Belgium) were used at a concentration of 0.75 µM. 3 µl of Fill-In product (total volume: 40 µl) were amplified using AccuPrime Pfx SuperMix (20 µl; Thermo Fisher Scientific, Waltham, Massachusetts, United States) in one PCR parallel (final reaction volume: 25 µl; final primer concentration: 200 nM each) thereby adding unique and sample-specific index combinations to the library molecules. Double indexing followed Kircher, Sawyer, and Meyer (2012)[17], but using index sequences from the NexteraXT index Kit v2 (Illumina). The number of PCR cycles was minimized and set to 12 cycles, to reduce the number of duplicates. The PCR temperature profile followed the manufacturer's recommendations but using an annealing temperature of 60°C, extending for 30 seconds during each cycle and performing a final elongation step for 5 minutes.

Purification during library preparation was conducted using the MinElute PCR Purification Kit (Qiagen, Hilden, Germany), while amplified libraries were purified with MSB Spin PCRapace (Invitek, Stratec Molecular, Berlin, Germany). Library concentrations were measured by Qubit Fluorometric quantitation (dsDNA HS assay, Invitrogen). The Agilent 2100 Bioanalyzer System (HS DNA, Agilent Technologies) was used to estimate fragment length distributions of the libraries. All negative controls were clear from contamination.

3.4. Sample screening

For all 24 samples, endogenous DNA preservation was determined using shallow shotgun sequencing to assess the quality of the extracts for deeper shotgun sequencing. Double-indexed libraries were diluted if necessary and pooled together in equimolar proportions according to their concentration measured by Qubit Fluorometric quantitation (dsDNA HS assay, Invitrogen). Screening runs were performed on the MiSeq sequencing platform (Illumina) at StarSEQ GmbH (Mainz, Germany) as single-end runs with 50 bp read length (Supplementary Dataset 1). Sequencing resulted in 500K to 1 million raw reads per sample (705358.5 raw reads on average), which were processed with the specific pipeline developed for aDNA analyses[18].

Raw files (FASTQ files) were demultiplexed by the sequencing facility. Adapter sequences were trimmed at the 3' end of each read according to Kircher (2012)[19], with criteria of at least 90% identity between adapter and read sequence, and a minimum adapter length of 1 bp. Reads with a base quality score below 15 in more than 5% of the bases of a sequencing read were removed from the dataset[19]. After quality check, reads were aligned to the human reference genome GRCh37/hg19 with the default parameters using BWAaln[20]. By checking the start and end of the reads, a number of duplicates was estimated and removed by the MarkDuplicates tool from the Picard tools package (picardtools, http:// broadinstitute.github.io/picard). The duplication rate (difference between unique reads and all reads aligned to the reference genome) is very informative as a high duplication rate leads to an overestimation of the endogenous content in the calculation. A high duplication rate also suggests low complexity and therefore low quality of the sample. In addition, reads were filtered for a minimum length of 30 bp.

The sample quality is evaluated by calculating the endogenous DNA content

after duplicate removal. The percentage of endogenous DNA was calculated as the ratio of unique aligned reads to the reference genome against the total number of reads after quality filtering, and it varied in range from 8% up to 70% (with only two samples below 20%) demonstrating a very good preservation of the petrous bones from the Mokrin necropolis (Supplementary Dataset 1).

Post-mortem damage patterns in aligned, length-filtered sequence reads were obtained with the software package MapDamage 2.0[21] for all the libraries to evaluate sample authenticity. The MapDamage program identifies the increase in C to T transitions at the ends of the reads, a damage pattern typical of ancient DNA. Due to relatively good biomolecular preservation of the Mokrin samples, deamination rates ranged from 0.13 to 0.26 at the first base at the fragment's 5' ends (Supplementary Dataset 1).

3.5. Sample preparation for whole-genome sequencing

Additional libraries were prepared for all 24 samples for deeper shotgun sequencing to obtain genome-wide coverage of ~1X per sample. Prior to library preparation the DNA extracts were treated with USER enzyme, which eliminates uracil bases caused by time dependent deamination of aDNA leaving a single nucleotide gap. 5 µl of USER enzyme (New England Biolabs, Ipswich, Massachusetts, United States) was added to 16.25 µl of DNA extract and the mixture was incubated for three hours at 37°C[22]. The blunt-end repair step followed immediately. The libraries for deep sequencing were also prepared with new unique index combinations to prevent the effect of index hopping during sequencing. Amplifications of all libraries were performed with AccuPrime Pfx SuperMix (Thermo Fisher Scientific, Waltham, Massachusetts, United States) as explained above (see *3.3. Library Preparation*) but in 12 PCR parallels to increase the complexity of the libraries. The number of cycles for the PCR reactions varied between samples (10 - 14 cycles) as it was adjusted to the presumed quantity of DNA fragments in the library. After amplification, all parallels of the same library were purified together and quantified as described above. For sequencing, libraries were pooled according to their concentrations measured on Qubit and taking into account the endogenous DNA content as estimated by MiSeq sequencing. Subsequently, the pooled libraries were purified with magnetic beads (Agencourt AMPure XP beads, Beckmann Coulter). The samples were sequenced on Illumina's NovaSeq 6000 sequencing platform (S2 flow cell, 50 bp, paired-end run) at the Next Generation Sequencing Platform (Institute of Genetics), University of Bern, Switzerland.

Supplementary information 4. Read Processing

Jens Blöcher, Joachim Burger

4.1. Sequence alignment

Residual adapters were removed from both read pairs prior to merging using trimmomatic 0.36[23], discarding reads shorter than 30 base pairs. Forward and reverse reads were collapsed into a single read, using BBMerge[24], requiring a minimum overlap of 3 bp. Only reads where the forward and reverse read could be combined were aligned against the reference genome (GRCh37/hg19) using bwa aln[20] with disabled seeding (options -l 1024 -n 0.02). During the conversion to the BAM format,

reads were filtered for a minimal mapping quality of 30. PCR duplicates were marked, using sambamba[25], prior to realignment with GATK[26] around known SNPs and InDels. Authenticity of the ancient DNA was established by determining deamination patterns using ATLAS[27].

4.2 Variant Detection

SNP-Calling was done following the approach described in Hofmanovà *et al.* (2016)[28], using the ATLAS package[27]. Genome-wide calls were obtained with the Maximum Likelihood approach described in Hofmanovà *et al.* (2016)[28], while majority-allele calls were performed for the SNPs overlapping the 1240k capture array described in Mathieson *et al.* (2015)[29] (Supplementary Dataset 1), the Y-chromosome and the mitochondrial chromosome. In each case, sequencing errors and PMD patterns were considered during variant detection.

4.3 Uniparental Markers

MT-Haplotypes were determined by uploading a combined sample vcf to the Haplogrep2.0 web application[30] (https://haplogrep.i-med.ac.at/app/index.html). For the Y-chromosomal haplotype assignment, yhaplo[31] was used. Therefore the majority-allele calls were transformed to the transposed plink format and then converted to the yhaplo specific input format. Determination was based on the isogg tree provided alongside the script (isogg.2016.01.04.txt).

4.4. Sex determination

Reads obtained by whole genome sequencing were used for biological sex determinatios, following the approach described in Skoglund *et al.* (2013)[32]. For three individuals the anthropological sex determination differed from molecular sexing (graves 122S, 220, 257B, see table 1). While the Skoglund *et al.* (2013) method[32] did not give a conclusive result for the individual 211, the Cassidy *et al.* (2020)[33] approach gave a clear result and identified the individual as a male, which is consistent with the morphological determination.

Supplementary information 5. Ancestry Analysis
Yoan Diekmann

5.1. Principal component analysis (PCA)

PCA was performed with LASER (version 2.04;[34]) following the approach described previously[28]. After generating a reference space of modern European individuals (data published as part of reference[35]); Southern European [Italian North/South, Spanish /North, Canary Islander, Maltese, Greek], Sicilian, Basque, Sardinian, Cypriot, Central- and Eastern European [Albanian, Bulgarian, Romanian, Hungarian, Czech, German, French], Croatian, Slavic [Russian, Ukrainian, Belarusian, Polish, Sorb, Mordovian], Baltic and Finnish [Estonian, Lithuanian, Finnish], British Isles [English, Orcadian, Scottish, Irish /Ulster, Shetlander], Icelandic, Norwegian), we projected the BAM files of the Mokrin individuals presented here into the reference space via Procrustes analysis implemented in LASER based on ten replicates (Supplementary Figure S3).

5.2. Pairwise comparison of ancestry proportions

We analytically compute the probability that an admixture proportion is greater than another by comparing the Normal distributions generated by *qpAdm* via $P(X > Y) = ½ \text{erfc}((\mu_y-\mu_x)/\sqrt{2(\sigma_x^2 + \sigma_y^2)})$, where *erfc* is the complementary error function (Supplementary Figure S4, S5; Supplementary Dataset 2).

5.3. f-statistics

All *f*-statistics, *i.e.* outgroup f_3, admixture f_4, and f_4 admixture proportions, were computed with *qp3Pop*, *qpDstat* in f_4 mode and *qpAdm* from the ADMIXTOOLS[36] package respectively, with default parameters and on the positions defined by the *HOIll* set of SNPs[37]. Modern reference individuals were first published in reference[38], and retrieved together with ancient reference samples from David Reich's lab website (https://reich.hms.harvard.edu/downloadable-genotypes-present-day-and-ancient-dna-data-compiled-published-papers; accessed on August 12, 2019). All *qp3Pop* and *qpDstat* analyses are performed with Khomani as outgroup, all *qpAdm* runs used the set of outgroups Han, Karitiana, Mbuti, Onge, Papuan, Mota, Ust'-Ishim, MA1, El Mirón, GoyetQ116-1, and admixture components Western hunter gatherers (Loschbour_snpAD.DG, LaBrana1_published.SG, I1507, Bichon.SG, Villabruna), Aegean Neolithic farmers (I1581, I1583, I1580, I1585, I1579, I1099, I1103, I1101, I1097, I0744, I1096, I1098, I0708, I0745, I0746, I0707, I0709, I0736, I0723, Bon002.SG, Tep003.SG, Bar31.SG, Bar8.SG), and Eastern European steppe-like (I0231, I0370, I0444, I0357, I0429, I0438, I0443, RISE547.SG, RISE548.SG, RISE550.SG, RISE552.SG). When Mokrin individuals were pooled to be analysed together, we excluded one from pairs of related individuals choosing the one with fewer covered SNPs, resulting in the set 237, 257A, 122E, 122S, 163, 186, 220, 223, 224, 246, 247, 260, 282, 287, 295, 302 (Supplementary Figure S5, S7).

Supplementary information 6. Kinship analyses

Krishna Veeramah

6.1. lcMLkin analysis using Mokrin allele frequencies

In the first approach we applied the approach of Lipatov *et al.* (2015)[39] using the C++ software lcMLkin, which utilizes genotype likelihoods in a maximum-likelihood framework to estimate diploid identity-by-descent probabilities and kinship coefficients. In this version underlying population allele frequencies were estimated using genotype calls from the Mokrin data itself. Two aspects complicate this usage. First, our data consist of low coverage sequencing, and thus there are few SNPs amongst our ~6 million that will be called (let alone reliably) across all 24 Mokrin individuals. Therefore we restricted our analysis to 467,179 SNPs where at least 70% of individuals were called. Generally this ensured there were ~200,000 usable sites for any pairwise sample comparison for lcMLkin. Secondly, the inclusion of related individuals will bias population allele frequency estimation. Therefore we performed one round of relatedness estimation using all samples, identified pairs of individuals with pi_hat > 0.05 and performed a second round such that allele frequencies were only estimated based on unrelated individuals based on this criteria (i.e. for a pair of related samples, one was not including in the allele frequency estimation). Following the second round of lcMLkin, nine pairwise comparisons consisting of 15 individuals

yielded pi_hat values > 0.08, while the rest were <=0.004, and thus likely unrelated.

6.2. lcMLkin analysis using 1000 Genome allele frequencies

In the second approach, we again used the genotype-likelihood maximum likelihood framework of lcMLkin. However, we further adapted the approach of Lipatov *et al.* (2015)[39] in three ways.

1) Use of population allele frequencies estimated from an external population. In this case we applied the allele frequencies estimated from 99 CEU individuals and 503 EUR individuals from the 1000 Genomes project[40]. In comparison to the first approach, these allele frequencies will be less accurate compared to the true underlying Mokrin population allele frequencies, but will be more precise as they are based on larger sample sizes. This will also increase the number of SNPs usable for each pairwise comparison (as they no longer need to be called in at least 70% of the Mokrin individuals).

2) If the true population allele frequencies of Mokrin and CEU/EUR are not that different ($F_{ST}<0.01$), then this should not greatly affect relatedness estimation using lcMLkin (see [39]). However, we developed an extension of lcMLkin that can further account for such distances by incorporating the identity-by-descent model of Anderson and Weir (2007)[41] (Table 2 of that paper). This allows us to a priori define an F_{ST} that accounts for drift between the population allele frequencies of the population being tested for relatedness versus those from the population from which allele frequencies are being estimated.

3) lcMLkin assumes that SNPs are independent, as log likelihoods for each SNP are summed across loci. It is possible to perform linkage disequilibrium (LD) pruning on the reference dataset (CEU or EUR) prior to analysis. However, this is inefficient when there is a lot of missing data in the population being tested (usually one of a pair of SNPs that show high correlations are randomly dropped from the analysis). Therefore we performed individual LD pruning of SNPs for every individual pairwise comparison using only SNPs that were called in that pair. LD pruning was performed using PLINK 2[42] using the command --indep-pairwise 50 5 0.2 for the underlying population used to estimate population allele frequencies.

Initially using both CEU and EUR population allele frequencies and an F_{ST} of 0.0, we identified the same nine pairs of related individuals from the first analysis with the highest pi_hat values. However, it was also noticeable that pi_hat for all other pairs was noticeably higher, with 166 (60%) and 54 (20%) pairs from a total of 276 pairs having pi_hat>=0.05 for CEU and EUR respectively. Therefore we reperformed the analysis assuming F_{ST} of 0.01, 0.02, 0.03. 0.04 and 0.05. Boxplots of all pi_hat values for each F_{ST} value show that overall pi_hat declines with increasing F_{ST}, essentially reaching a valley of 0 for both CEU and EUR at 0.03, consistent with most individuals being unrelated in the first analysis using Mokrin allele frequencies and this being the appropriate F_{ST} to use (Supplementary Figure S8, S9). Under this criteria all nine individuals from the previous analysis were considered related with similar pi_hat values, as well as one extra pair potentially being marginally related (individuals buried in graves 223 and 260, pi_hat = 0.073 and 0.064).

6.3. READ analysis

As an alternative to lcMLkin we utilized the approach of Kuhn, Jakobsson and Günther (2018)[43] implemented in the software READ, because it does not rely on underlying population allele frequencies and can work with very low coverages and may therefore have additional power for our dataset. READ relies on a normalization

that assumes most individuals are unrelated. Therefore we applied this method to pseudo-haploid calls generated for the Mokrin data as well as for the 99 unrelated CEU individuals. We used a subset of the SNPs from the analysis above where the minor allele frequency was at least 5% in the CEU samples (5,496,079 SNPs). Utilizing this approach, the same 9 pairs of individuals as in the previous analysis were identified, either being first or second degree related. All other pairs of individuals were considered unrelated using this method (Supplementary Figure S10).

Summary

Nine pairs of individuals (out of a total of 15 individuals) were identified as showing evidence of significant genetic relatedness. One pair showed marginal evidence using the second approach (using external population allele frequencies but was not replicated in the other two). The estimated degree of relatedness from the READ analysis agreed with that based on the kinship coefficients from the lcMLkin analysis. A summary of significant pairs of the combined analysis can be found in Table 2.

Supplementary information 7. Population Structure

Jens Blöcher, Joachim Burger, Yoan Diekmann

7.1. Inbreeding coefficient F

We used ATLAS[27] to estimate the inbreeding coefficient F (task=inbreeding) to detect potential population structure among the Mokrin samples, as described in Burger *et al.* (2020)[44]. In a structured population F is expected to be positive, due to a deficit of heterozygous genotypes, as described by the Wahlund effect.

Only variants with a quality >= 40 that could be determined in ten or more samples were used in this analysis. The MCMC was run for 10^6 iterations, after ten initial burn-ins of 500 runs each. This resulted in an inbreeding coefficient of F=0, consistent with a lack of structure. To further exclude the assumption of a structured population, we re-ran the analysis, but this time forcing F>0. This resulted in a mean F of 1.3222e-05 +- 1.2447e-05.

7.2. F_{ST} between Mokrin Samples

We compiled a data set of 1092 SNPs that were covered at least twice in all Mokrin samples with a genotype quality of at least 30, that had a minor allele frequency of at least 0.05 in the 1000 Genomes CEU population to calculate Hudsons's F_{ST} between subgroups of samples. When partitioning the samples into "northern" and "southern" groups based on the PCA plot, as well as samples with and without (211, 247, 257B, 295) hunter-gatherer ancestry in the qpAdm analysis (Supplementary information 5), a F_{ST} of zero was estimated.

Supplementary information 8. Functional Markers

Jens Blöcher

Allele frequencies were estimated as described in Burger *et al.* (2020)[44]. The ATLAS software[27] was used to determine genotype likelihoods for each sample, while first learning the distribution of the postmortem-damage patterns and sequencing errors for each sample individually. Based on the individual genotype likelihoods, population wide frequencies were estimated, using the doBaysian option, that also estimates corresponding 90% credible intervals. Differences to frequencies in modern day populations for the functional markers described in Mathieson *et al.* (2015)[29] and Veeramah *et al.* (2018)[45], were examined by calculating F_{ST} values, using Hudson's estimator as described in Bhatia *et al.* (2013)[46], using a custom script. In absence of a direct reference population from the region of origin of the Mokrin samples, frequencies were compared to the European populations of the 1000 Genomes project[47] excluding the Finish (FIN). A list of SNPs for which a F_{ST} in the top 99th quantile of resulting values was found is reported in an excel file (Supplementary Dataset 3. Frequencies_Fsts.xlsx), for each of the four European populations.

Supplementary information references

1. Girić, M. *Mokrin. Nekropola ranog bronzanog doba: Mokrin. The early bronze age necropolis*. (Dissertationes et monographie XI. Washington, Kikinda i Beograd: Smithsonian Institution, Narodni muzej, Arheološko društvo Jugoslavije, Jugoslavija, 1971).
2. O'Shea, J. M. *Villagers of the Maros: A Portrait of an Early Bronze Age Society*. (Plenum Press, New York, NY, 1996).
3. O'Shea, J. A radiocarbon-based chronology for the Maros Group of southeast Hungary. *Antiquity* vol. **66**, 97–102 (1992).
4. Stefanović, S. *Skeletal markers of occupational stress in later prehistory: Mokrin necropolis (2000–1800 B.C.)*. (University of Belgrade, Belgrade, Serbia, 2008).
5. Bökönyi, S. Životinjski ostaci iz grobova nekropole bronzanog doba u Mokrinu. in *Mokrin II: nekropola ranog bronzanog doba, Dissertationes et monographie XII* (ed. Foltiny, S.) 91 – 96 (Washington, Kikinda i Beograd: Smithsonian Institution, Narodni muzej, Arheološko društvo Jugoslavije, Jugoslavija, 1972).
6. Farkas, G. & Liptak, P. Antropološko istrazivanje nekropole u Mokrinu iz ranog bronzanog doba. in *Mokrin: nekropola ranog bronzanog doba, Dissertationes et monographie XI* (ed. Girić, M.) 239–271 (Washington, Kikinda i Beograd: Smithsonian Institution, Narodni muzej, Arheološko društvo Jugoslavije, Jugoslavija, 1971).
7. Rega, E. A. Biological Correlates of Social Structure in the Early Bronze Age Cemetery at Mokrin. (University of Chicago, USA, 1995).
8. Buikstra, J. E. & Ubelaker, D. H. *Standards for data collection from human skeletal remains*. (Arkansas Archeological Survey, Fayetteville, 1994).
9. Trotter, M. J. Estimation of stature from intact long bones. in *Personal Identification in Mass Disasters* (ed. Stewart, T. D.) 71–83 (Smithsonian Institution Press, Washington, DC, 1970).
10. Porčić, M. & Stefanović, S. Physical activity and social status in Early Bronze Age society: The Mokrin necropolis. *Journal of Anthropological Archaeology*

vol. **28,** 259–273 (2009).
11. Ljuština, M., Krečković, M. & Radišić, T. Notes on Columbella Shells from the Bronze Age Necropolis Mokrin, Northern Serbia. in *Homage to Prof. dr Cristian Schuster on his 60th Anniversary* (ed. Comsa, A.) (Maiastra Publishing House, Targu Jiu, 2019).
12. Scheu, A. *et al.* The genetic prehistory of domesticated cattle from their origin to the spread across Europe. *BMC Genet.* **16**, 54 (2015).
13. Gamba, C. *et al.* Genome flux and stasis in a five millennium transect of European prehistory. *Nature Communications* vol. **5** (2014).
14. Hansen, H. B. *et al.* Comparing Ancient DNA Preservation in Petrous Bone and Tooth Cementum. *PLoS One* **12**, e0170940 (2017).
15. Yang, D. Y., Eng, B., Waye, J. S., Christopher Dudar, J. & Saunders, S. R. Improved DNA extraction from ancient bones using silica-based spin columns. *American Journal of Physical Anthropology* vol. **105**, 539–543 (1998).
16. MacHugh, D. E., Edwards, C. J., Bailey, J. F., Bancroft, D. R. & Bradley, D. G. The extraction and analysis of ancient DNA from bone and teeth: A survey of current methodologies. *Anc. Biomol.* **3**, 81–102 (2000).
17. Kircher, M., Sawyer, S. & Meyer, M. Double indexing overcomes inaccuracies in multiplex sequencing on the Illumina platform. *Nucleic Acids Res.* **40**, e3 (2012).
18. Sell, C. Addressing challenges of ancient DNA sequence data obtained with next generation methods. (University of Mainz, Germany, 2017).
19. Kircher, M. Analysis of High-Throughput Ancient DNA Sequencing Data. in *Ancient DNA: Methods in Molecular Biology (Methods and Protocols)* (ed. Beth Shapiro And) vol. 840 197–228 (Humana Press, New York, NY, 2012).
20. Li, H. & Durbin, R. Fast and accurate short read alignment with Burrows-Wheeler transform. *Bioinformatics* vol. **25,** 1754–1760 (2009).
21. Jónsson, H., Ginolhac, A., Schubert, M., Johnson, P. L. F. & Orlando, L. mapDamage2.0: fast approximate Bayesian estimates of ancient DNA damage parameters. *Bioinformatics* vol. **29,** 1682–1684 (2013).
22. Verdugo, M. P. *et al.* Ancient cattle genomics, origins, and rapid turnover in the Fertile Crescent. *Science* **365**, 173–176 (2019).
23. Bolger, A. M., Lohse, M. & Usadel, B. Trimmomatic: a flexible trimmer for Illumina sequence data. *Bioinformatics* **30**, 2114–2120 (2014).
24. Bushnell, B., Rood, J. & Singer, E. BBMerge - Accurate paired shotgun read merging via overlap. *PLoS One* **12**, e0185056 (2017).
25. Tarasov, A., Vilella, A. J., Cuppen, E., Nijman, I. J. & Prins, P. Sambamba: fast processing of NGS alignment formats. *Bioinformatics* **31**, 2032–2034 (2015).
26. McKenna, A. *et al.* The Genome Analysis Toolkit: A MapReduce framework for analyzing next-generation DNA sequencing data. *Genome Research* vol. **20**, 1297–1303 (2010).
27. Link, V. *et al.* ATLAS: Analysis Tools for Low-depth and Ancient Samples. (2017) doi:bioRxiv 10.1101/105346.
28. Hofmanová, Z. *et al.* Early farmers from across Europe directly descended from Neolithic Aegeans. *Proc. Natl. Acad. Sci. U. S. A.* **113**, 6886–6891 (2016).
29. Mathieson, I. *et al.* Genome-wide patterns of selection in 230 ancient Eurasians. *Nature* **528**, 499–503 (2015).
30. Weissensteiner, H. *et al.* HaploGrep 2: mitochondrial haplogroup classification in the era of high-throughput sequencing. *Nucleic Acids Res.* **44**, W58–63 (2016).

31. Poznik, G. D. Identifying Y-chromosome haplogroups in arbitrarily large samples of sequenced or genotyped men. (2016) doi:bioRxiv 10.1101/088716.
32. Skoglund, P., Storå, J., Götherström, A. & Jakobsson, M. Accurate sex identification of ancient human remains using DNA shotgun sequencing. *Journal of Archaeological Science* vol. **40**, 4477–4482 (2013).
33. Cassidy, L. M. *et al.* A dynastic elite in monumental Neolithic society. *Nature* **582**, 384–388 (2020).
34. Wang, C., Zhan, X., Liang, L., Abecasis, G. R. & Lin, X. Improved Ancestry Estimation for both Genotyping and Sequencing Data using Projection Procrustes Analysis and Genotype Imputation. *The American Journal of Human Genetics* vol. **96**, 926–937 (2015).
35. Lazaridis, I. *et al.* Genomic insights into the origin of farming in the ancient Near East. *Nature* **536**, 419–424 (2016).
36. Patterson, N. *et al.* Ancient admixture in human history. *Genetics* **192**, 1065–1093 (2012).
37. Haak, W. *et al.* Massive migration from the steppe was a source for Indo-European languages in Europe. *Nature* **522**, 207–211 (2015).
38. Mallick, S. *et al.* The Simons Genome Diversity Project: 300 genomes from 142 diverse populations. *Nature* **538**, 201–206 (2016).
39. Lipatov, M., Sanjeev, K., Patro, R. & Veeramah, K. R. Maximum Likelihood Estimation of Biological Relatedness from Low Coverage Sequencing Data. *bioRxiv* 023374 (2015) doi:10.1101/023374.
40. 1000 Genomes Project Consortium *et al.* An integrated map of genetic variation from 1,092 human genomes. *Nature* **491**, 56–65 (2012).
41. Anderson, A. D. & Weir, B. S. A Maximum-Likelihood Method for the Estimation of Pairwise Relatedness in Structured Populations. *Genetics* vol. **176**, 421–440 (2007).
42. Chang, C. C. *et al.* Second-generation PLINK: rising to the challenge of larger and richer datasets. *Gigascience* **4**, 7 (2015).
43. Kuhn, J. M. M., Jakobsson, M. & Günther, T. Estimating genetic kin relationships in prehistoric populations. *PLOS ONE* vol. 13 e0195491 (2018).
44. Burger, J. *et al.* Low Prevalence of Lactase Persistence in Bronze Age Europe Indicates Ongoing Strong Selection over the Last 3,000 Years. *Curr. Biol.* (2020) doi:10.1016/j.cub.2020.08.033.
45. Veeramah, K. R. *et al.* Population genomic analysis of elongated skulls reveals extensive female-biased immigration in Early Medieval Bavaria. *Proc. Natl. Acad. Sci. U. S. A.* **115**, 3494–3499 (2018).
46. Bhatia, G., Patterson, N., Sankararaman, S. & Price, A. L. Estimating and interpreting FST: The impact of rare variants. *Genome Research* vol. **23**, 1514–1521 (2013).
47. The 1000 Genomes Project Consortium & Auton, A., Brooks, L.D., Durbin, R.M., Garrison, E.P., Kang, H.M., Korbel, J.O.,Marchini, J.L., McCarthy, S., McVean, G.A., and Abecasis, G.R. A global reference for human genetic variation. *Nature* **526**, 68–74 (2015).

Legends for Supplementary Figures following the document:

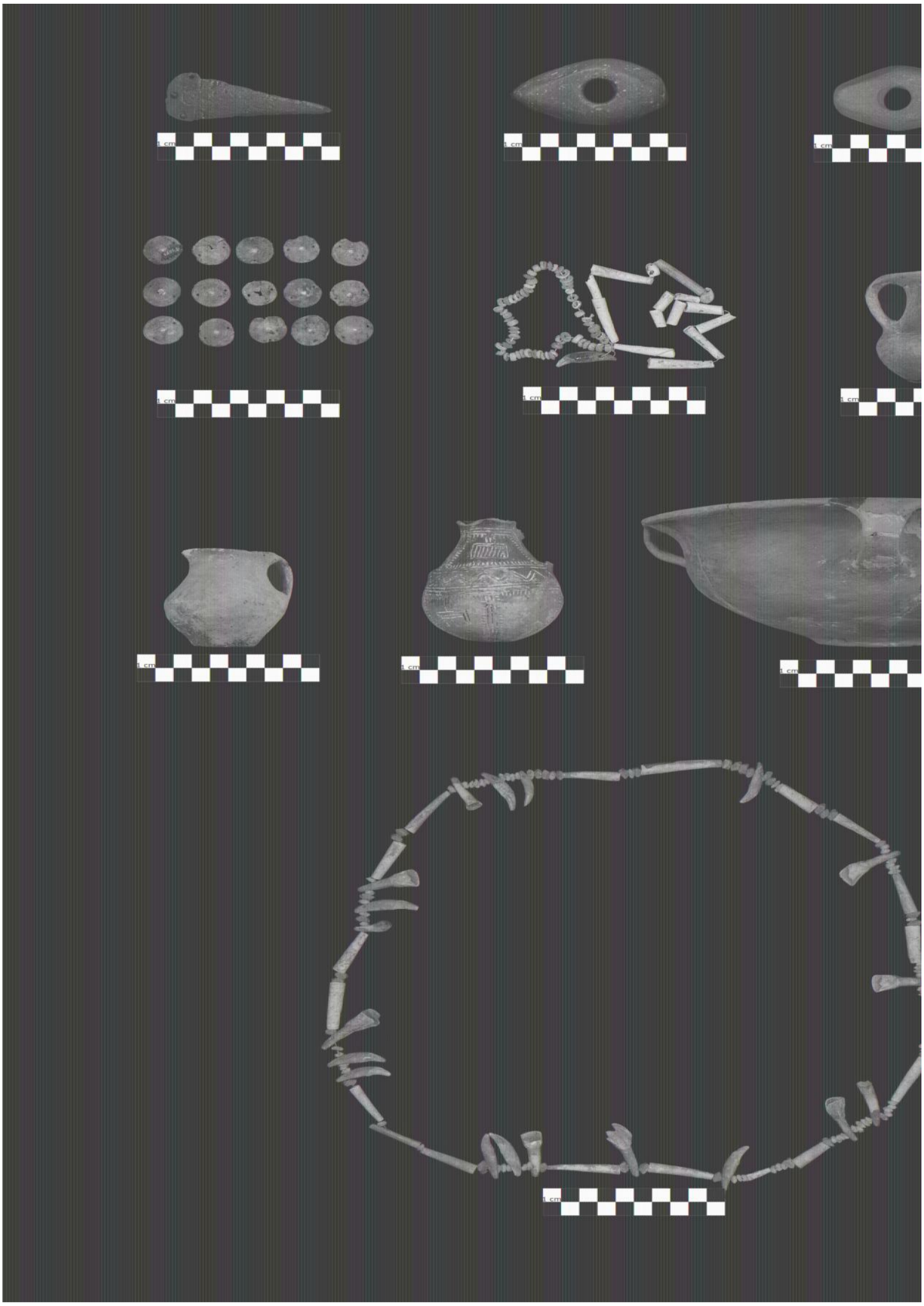

Supplementary Figure S1. Grave goods found with the sampled individuals. a. dagger (grave 211), b. axe (grave 163), c. axe (grave 243), d. head ornament (grave 161), e. necklace (grave 122S), f. beaker (grave 181), g. beaker (grave 163), h. beaker with white encrustation (grave 282), i. bowl (grave 163), j. beaded sash (grave 161).

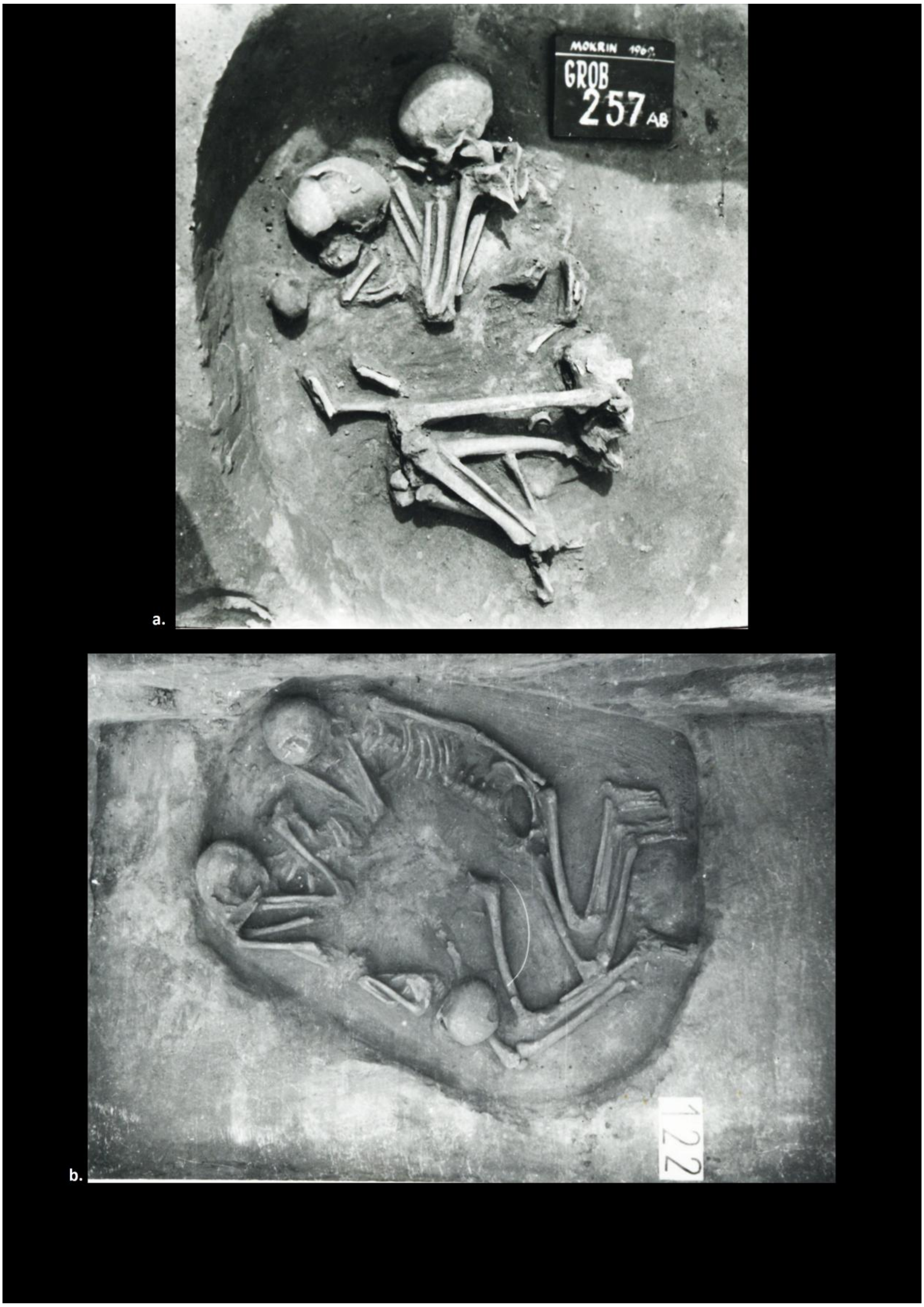

Supplementary Figure S2. Multiple burials (a. double burial, b. triple burial)

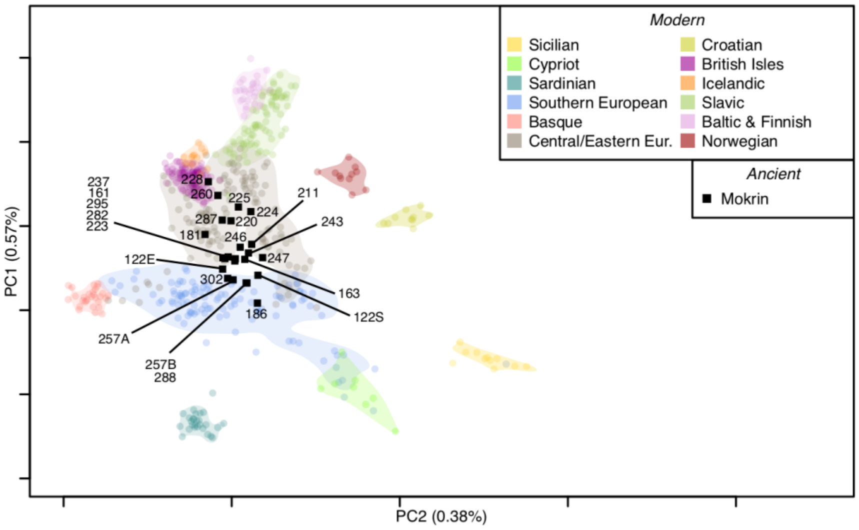

Supplementary Figure S3. **PCA projecting Mokrin individuals onto modern populations from northern, eastern and southern Europe**.

We observe variation amongst Mokrin individuals at the level of the first two principal components. This may surprise given the degree of relatedness between the individuals, however, the relative spread is likely an artefact of dimensionality reduction and projection as the inferred kinship relations are reflected correctly for example in the f_3 statistics in Supplementary Figure S6. All samples clearly fall within modern European genetic variation, specifically onto modern Central/Eastern (in the middle of Bulgaria, Romania, Hungary, Germany, France) and Southern Europeans (Italians).

Abbreviations: principal component (PC)

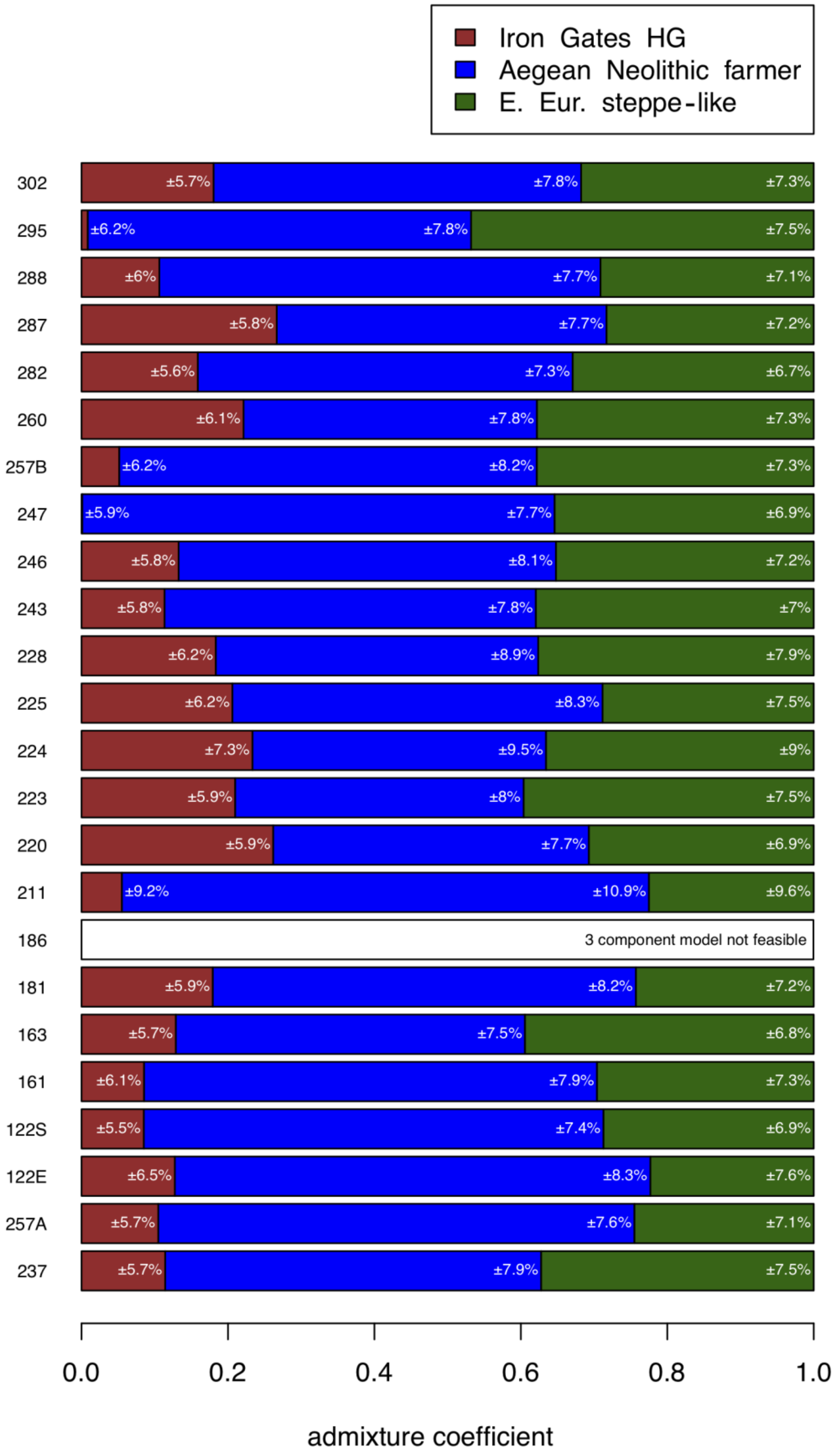

Supplementary Figure S4. **Individual admixture proportions.**
All Mokrin individuals but 186 can be modelled as a mixture of three genomic ancestry components. However, note that the individuals buried in the graves 211, 247, 257B and 295 lack statistical support for a three ancestry component model over a simpler two component

model without hunter-gatherers (as indicated by *qpAdm* by higher tail probability and non-significant p-value for the nested model). In Supplementary Figure S7 we tested if some hunter gatherers show higher affinities to the Mokrin individuals than others and would therefore be more adequate as HG base component, but found no consistent difference apart from the La Braña. We therefore chose Iron Gates HG, as they are geographically closest. Pairwise comparison of Eastern European steppe-like proportions finds no pair of individuals with statistically significant difference, see Supplementary Dataset 2. *Abbreviations:* hunter gatherer (HG), Eastern European (E. Eur.)

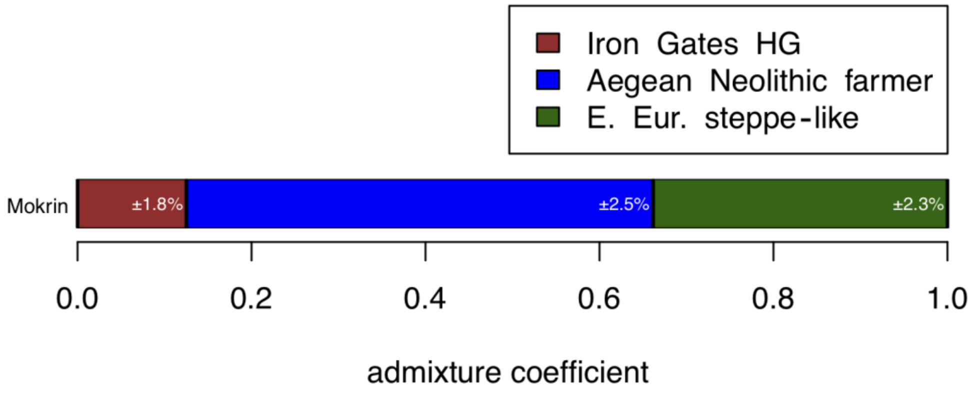

Supplementary Figure S5. Pooled individuals' admixture proportions.
Estimate of proportions of genomic ancestry components for the pooled set of Mokrin individuals: Iron Gates hunter gatherers 12.5%, Aegean Neolithic farmers 53.7%, Eastern European steppe-like 33.8%. *Abbreviations:* hunter gatherer (HG), Eastern European (E. Eur.)

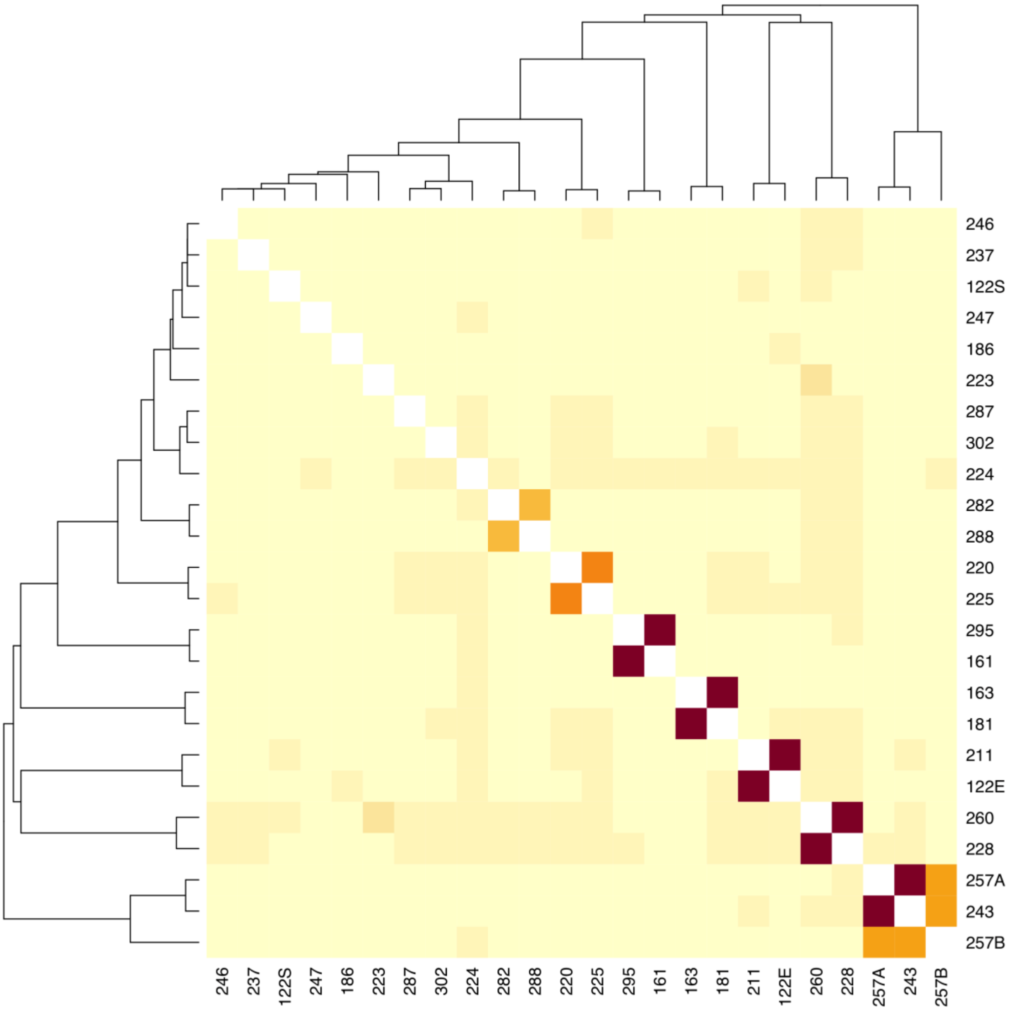

Supplementary Figure S6. **Heatmap of outgroup f_3 statistic between pairs of Mokrin individuals.**

Hierarchical clustering of vectors of outgroup f_3 statistics reflects the previously inferred kinship relations between some pairs of individuals.

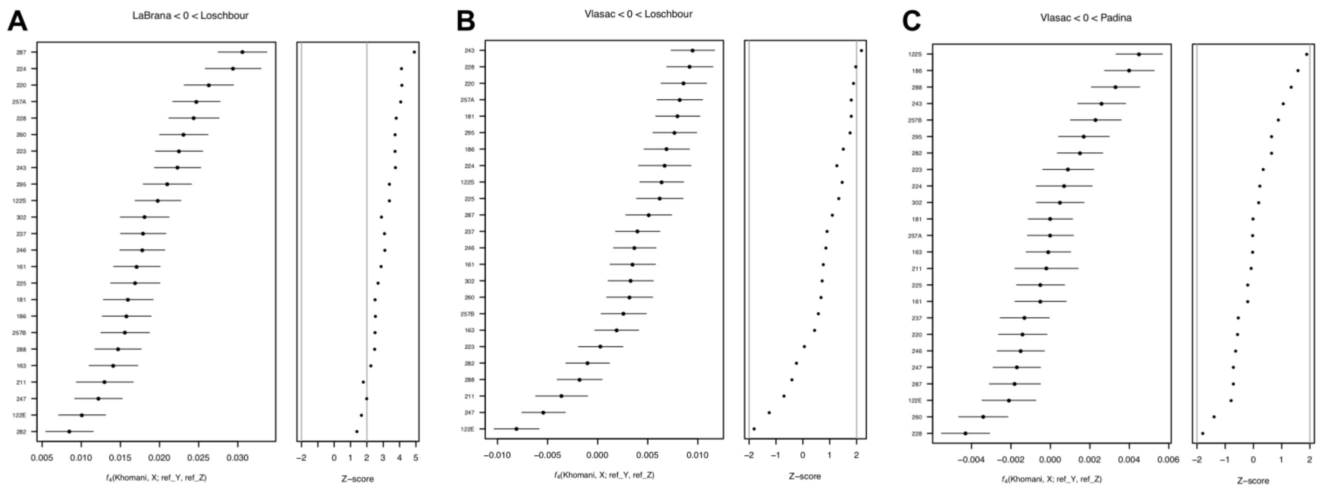

Supplementary Figure S7. A-C. **Admixture *f_4* between Mokrin and various pairs of hunter gatherers.**

Pooled Mokrin individuals have genetic affinity to the Loschbour hunter gatherer from Luxembourg over the Iberian La Braña (A). However, there is no affinity for the local Iron Gates hunter gatherers over Loschbour (B) or between Iron Gates hunter gatherers from different sites (C; Vlasac: I4873, I4874, I4875, I4876, I4877, I4878, I4880, I4881; Padina: I5232, I5233, I5235, I5236, I5237, I5238, I5239, I5240, I5242, I5244).

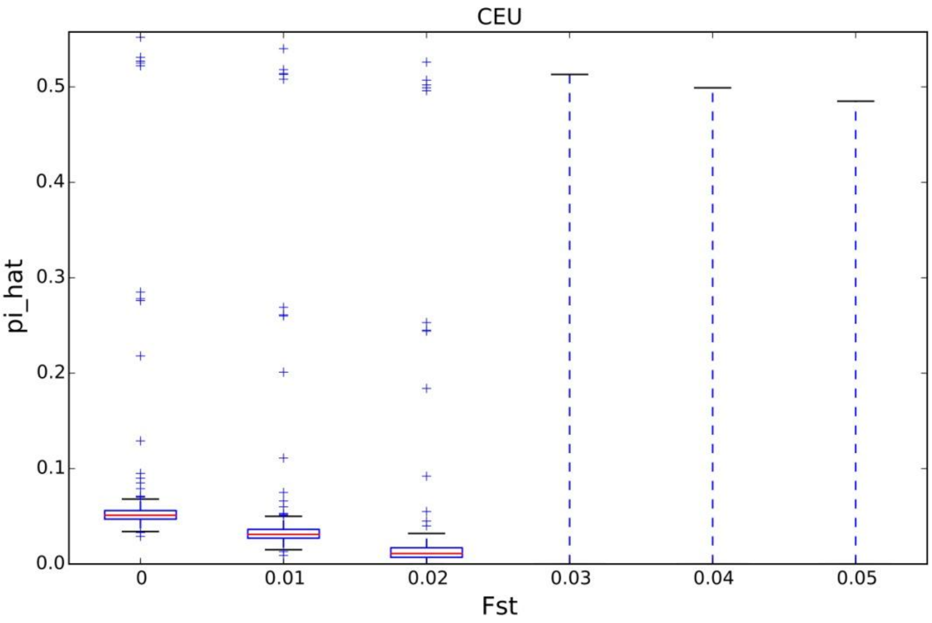

Supplementary Figure S8. Boxplot of pairwise pi_hat values for Mokrin samples using CEU allele frequencies and a range of F_{ST} to account for drift from the true frequencies.

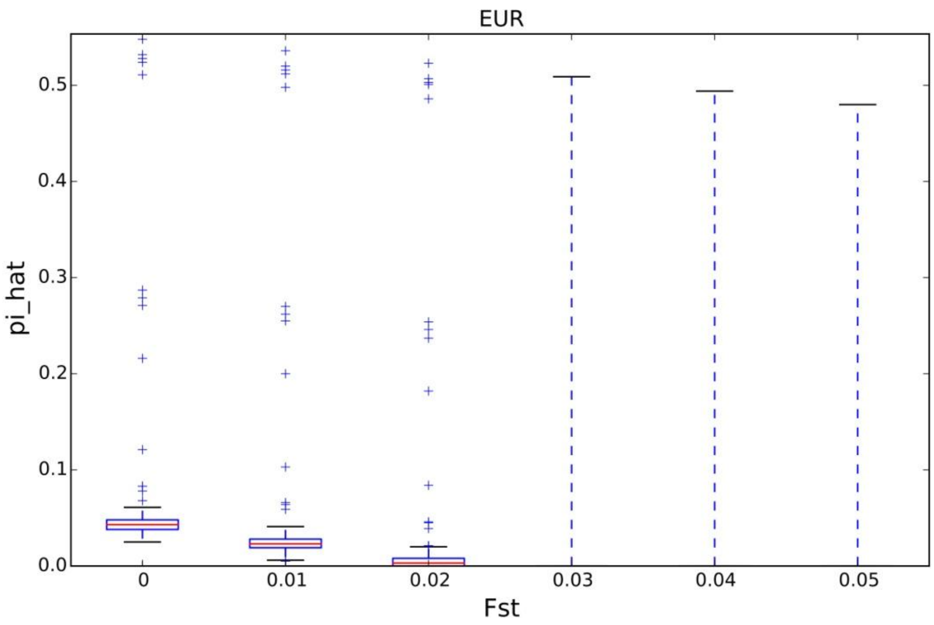

Supplementary Figure S9. Boxplot of pairwise pi_hat values for Mokrin samples using EUR allele frequencies and a range of F_{ST} to account for drift from the true frequencies.

Supplementary Figure S10. Kinship analyses on the Mokrin individuals performed by READ software.

Supplementary Table S1. Calibrated ^{14}C dates for the Mokrin necropolis.

Lab number	Dating material	Context	Uncalibrated (b.p.)	SD	Calibrated date (B.C.)	1 σ range
GrN-8809	Collagen	Grave 259	3500	35	1807	1737 -1879 B.C.
GrN-14181	Collagen	Grave 237	3595	35	1922	1831-2010 B.C.
GrN-14180	Collagen	Grave 227	3650	35	1992	1927-2107 B.C.
GrN-7977	Collagen	Grave 52	3650	50	1990	1920-2130 B.C.
GrN-14178	Collagen	Grave 110	3655	30	1991	1938-2108 B.C.
GrN-14179	Collagen	Grave 208	3690	30	2086	1974-2134 B.C.

Source - Table is modified from O'Shea (1992)[3].

Supplementary Table S2. Anthropological analysis of 24 individuals from the Mokrin necropolis.

Burial	Sex	Age	Height	Caries	LEH	C. orbitalia	P. hyperostosis	Periostitis
122E	M	6-9	N/A	no	no	present	absent	present
122S	F	35-50	N/A	no	yes	N/A	N/A	absent
161	F	9-11	N/A	no	no	absent	absent	absent
163	M	45-55	164.67	no	no	absent	absent	absent
181	F	>18	N/A	N/A	N/A	absent	absent	absent
186	F	8-11	N/A	no	no	absent	present	absent
211	M	50-55	N/A	no	no	absent	absent	present
220	M	15-25	163.01	no	no	present	absent	present
223	F	7-10	N/A	no	yes	absent	absent	absent
224	F	25-40	143.4	yes	no	absent	absent	absent
225	M	25-35	165.77	no	no	absent	absent	absent
228	F	35-50	155.52	yes	no	present	absent	absent
237	F	15-20	N/A	no	yes	absent	absent	present
243	M	20-35	162.5	no	no	N/A	N/A	present
246	F	45-50	154.84	yes	no	N/A	N/A	absent
247	F	10-12	N/A	no	yes	present	present	absent
257A	F	40-60	N/A	no	no	absent	absent	absent
257B	M	child	N/A	no	no	absent	absent	absent
260	M	15-18	N/A	no	no	N/A	absent	absent
282	M	15-20	N/A	yes	yes	N/A	N/A	present
287	F	20-35	155.29	no	no	absent	absent	absent
288	F	60+	N/A	yes	yes	absent	present	absent
295	M	15-20	N/A	no	yes	absent	absent	present
302	F	20-35	N/A	no	no	absent	absent	absent

Abbreviations: N/A - non applicable; LEH - *Linear enamel hypoplasia*; C.orbitalia - *Cribra orbitalia*; P.hyperostosis – *Porotic hyperostosis*

Supplementary Table S3. Alternative funerary treatments documented at the Mokrin necropolis.

Treatment			Number of burials
Alternative posture	A fully extended burial posture	It is possible that smaller bodies of infants and children lessened the need for flexion as the dimensions of the graves were constructed for adults.	1
	"rhomboidal" posture	body is placed on its back, with the legs strongly flexed at the knees, but with the legs spread apart	3
	"partial" postures	body is still roughly flexed, but it is rotated such that it lies on either its back or its front	4
Multiple burial			4
Symbolic burial			5
Cremation		probably from the earlier period of necropolis	6
Hearth burial			0
Urn burial			0
Mutilation of hands or feet, or both		postmortem modification found in roughly equal proportions among adults and children, but significantly more among males than females	41
Trepanation			7

Source - Table modified from O'Shea (1996)[2].

Other supplementary materials for this manuscript include the following:

Supplementary Dataset 1-3

Legends for separate files:

Supplementary Dataset 1. Sample preparation and read processing.xlsx

Supplementary Dataset 2. Pairwise comparison of Eastern European steppe-like ancestry proportions.
No pair has a probability of a different Eastern European steppe-like ancestry proportion above 95%. See Supplementary Materials and Methods for details on the computation. We exclude individual 186 as it has no feasible fit of a three components model.

Supplementary Dataset 3. Frequencies_Fsts.xlsx
